# Influence of Oxidative Stress on Time-Resolved Oxygen Detection by [Ru(Phen)_3_]^2+^ In Vivo and In Vitro

**DOI:** 10.3390/molecules26020485

**Published:** 2021-01-18

**Authors:** Veronika Huntosova, Denis Horvath, Robert Seliga, Georges Wagnieres

**Affiliations:** 1 Center for Interdisciplinary Biosciences, Technology and Innovation Park, P.J. Safarik University in Kosice, Jesenna 5, 041 54 Kosice, Slovakia; denis.horvath@upjs.sk (D.H.); robert.seliga@upjs.sk (R.S.); 2 Laboratory for Functional and Metabolic Imaging, Institute of Physics, Swiss Federal Institute of Technology in Lausanne (EPFL), Station 6, Batiment de Chimie, 1015 Lausanne, Switzerland; georges.wagnieres@epfl.ch

**Keywords:** oxygen detection, dendrogram analysis, hierarchical clustering, cancer cells, luminescence lifetime, hydrogen peroxide, oxidative stress, photodynamic therapy, time-resolved imaging

## Abstract

Detection of tissue and cell oxygenation is of high importance in fundamental biological and in many medical applications, particularly for monitoring dysfunction in the early stages of cancer. Measurements of the luminescence lifetimes of molecular probes offer a very promising and non-invasive approach to estimate tissue and cell oxygenation in vivo and in vitro. We optimized the evaluation of oxygen detection in vivo by [Ru(Phen)_3_]^2+^ in the chicken embryo chorioallantoic membrane model. Its luminescence lifetimes measured in the CAM were analyzed through hierarchical clustering. The detection of the tissue oxygenation at the oxidative stress conditions is still challenging. We applied simultaneous time-resolved recording of the mitochondrial probe MitoTracker^TM^ OrangeCMTMRos fluorescence and [Ru(Phen)_3_]^2+^ phosphorescence imaging in the intact cell without affecting the sensitivities of these molecular probes. [Ru(Phen)_3_]^2+^ was demonstrated to be suitable for in vitro detection of oxygen under various stress factors that mimic oxidative stress: other molecular sensors, H_2_O_2_, and curcumin-mediated photodynamic therapy in glioma cancer cells. Low phototoxicities of the molecular probes were finally observed. Our study offers a high potential for the application and generalization of tissue oxygenation as an innovative approach based on the similarities between interdependent biological influences. It is particularly suitable for therapeutic approaches targeting metabolic alterations as well as oxygen, glucose, or lipid deprivation.

## 1. Introduction

Tissue oxygenation is closely connected with the cell’s metabolic activity. In particular, oxygen supply reflects the pathophysiology of cancer cells. Several approaches to assess the tissue oxygenation were developed. One minimally invasive method to assess the level of oxygen is the time-resolved detection of oxygen-sensitive probes [1,2,3].

Dichlorotris(1,10-phenanthroline)-ruthenium(II) hydrate ([Ru(Phen)_3_]^2+^) is an interesting molecule emitting a bright luminescence. This relatively small molecule (700 Da) is sensitive to oxygen in solutions, in cells, and in vivo [4,5,6]. The luminescence lifetime of [Ru(Phen)_3_]^2+^ is quenched by oxygen, a mechanism that is governed by the Stern-Volmer relation [3]. This hydrophilic molecule is biocompatible and has low phototoxicity when it is localized in the extracellular space [3,7,8]. It takes a long time (24 h) for [Ru(Phen)_3_]^2+^ to accumulate in cells [7]. However, straight nuclear localization of [Ru(Phen)_3_]^2+^ was observed in damaged cells due to the increased permeability of their membrane [7,9]. Interaction with DNA increases the [Ru(Phen)_3_]^2+^ luminescence intensity as well as its luminescence lifetimes [10,11]. Therefore, [Ru(Phen)_3_]^2+^ is considered as an interesting oxygen-sensitive molecule, mainly to probe the oxygen level in extracellular compartments and solutions.

The concentration of oxygen inside and outside blood vessels can vary from place to place, and it can cause inaccuracies in determining the oxygen content. Recent trends suggest that a consistent interpretation of the measurements can be achieved not only by statistical evaluation but particularly by data classification. In general, it can be considered as a form of unsupervised learning [12,13] that is a form of learning leading to self-organized data clustering without previous labeling of the collected data by human experts. It is also important for the approach we present in this article to have a visualization that helps to understand the data. A specific application we present in this work is the use of clustering to facilitate the interpretation of [Ru(Phen)_3_]^2+^ luminescence lifetime data obtained in a pre-clinical in vivo model, the chicken embryo chorioallantoic membrane (CAM). We used hierarchical cluster analysis, which, in our specific conditions, involves the processing of data that reflect fluctuating oxygen levels, including fluctuations of the [Ru(Phen)_3_]^2+^ luminescence lifetimes, similarity aspects [Ru(Phen)_3_]^2+^ luminescence lifetime statistics, as well as spatial heterogeneities of the [Ru(Phen)_3_]^2+^ luminescence lifetimes induced by vascular heterogeneity.

Imbalance in oxygen supply and consumption may induce oxidative stress resulting from the production of reactive oxygen species (ROS) in cells. ROS are created as inherent products of oxygen metabolism in cells. Generally, the level of oxidative stress in cells is related to the amount of protein, lipids and DNA damages, and antioxidant status [14]. Recently, we have employed time-resolved imaging based on MitoTracker^TM^ Orange CMTMRos (MTO) to evaluate oxidative stress-induced in mitochondria of cancer cells [15]. This approach allows us to detect low-level oxidative stress-induced in cells. MTO is a mitochondrial probe which, besides oxidative stress visualization, enables to assess the integrity of mitochondria and to measure the mitochondrial membrane potential [16].

It is time and cost-saving, and very informative to perform multimodal visualization and simultaneous detection of several parameters, such as the level of oxygen and oxidative stress, in the studied system. Several approaches based on the intensity and time-resolved microscopy were applied for dual molecular sensors detection of oxidative stress, mitochondria integrity, thiophenols, and oxygen consumption by genetically encoded photosensitizers (PSs) [17,18,19,20,21,22,23]. Recently, metal complexes were developed to detect oxygen consumption, enabling mitochondria targeting and tracking morphological changes of mitochondria [24,25,26].

It is of high importance to eliminate the photodamages generated during oxygen and oxidative stress detection. Virtually, all fluorescence/phosphorescence molecular probes are more or less potent PSs. Their fluorescence and phosphorescence are often excited with laser light at wavelengths corresponding to maxima of the probe’s absorption. Excited probes/molecules stay in their triplet states during times ranging typically between 100 ns and 100 µs. These relatively long times are sufficient to enable an interaction of the probes with other molecules and biomolecules like proteins and lipids. Moreover, in the presence of molecular oxygen, such photoreaction can lead to oxidation and/or peroxidation of proteins and lipids. The initiation of these photoreactions strongly depends on the probes/molecule concentration and of the fluence rate generated by the light source. The photoreaction in cells may produce oxidative stress, ROS, and global photodamages in cells, which can lead to a decrease of their proliferation. The mechanisms described above are involved in photodynamic therapy (PDT), a treatment modality, in which light, PSs, and oxygen interplay together [27]. Ideal PS for PDT should be selective enough to generate photodamages only in the lesions to be treated. In contrast to PSs, ideal oxygen and oxidative stress-sensitive fluorescence and phosphorescence probes should be minimally phototoxic to the probed tissues.

The spectral properties of MTO and [Ru(Phen)_3_]^2+^ interfere with spectral properties of clinically applied PSs [27]. To demonstrate the sensitivity of MTO and [Ru(Phen)_3_]^2+^ during PDT, we have chosen curcumin with promising spectral and PDT properties [28,29]. Curcumin is a natural polyphenolic compound extracted from Curcuma longa which increases or decreases oxidative stress according to the condition in which it is present [30]. The phototoxicity of curcumin is ascribed to singlet oxygen and superoxide radicals production [28,29]. However, singlet oxygen scavenging by curcumin was reported as well [31]. Due to curcumin spectral properties and its biological activity, curcumin represents an interesting molecule in PDT.

In the present work, the main aim was to optimize the evaluation of oxygen detection in vivo by [Ru(Phen)_3_]^2+^. This is why we decided to apply the cluster analysis of the [Ru(Phen)_3_]^2+^ luminescence lifetimes measured in the CAM for this reason. The detection of the tissue oxygenation at an oxidative stress condition is still challenging. Therefore, we have suggested applying time-resolved recording of MTO-FLIM and [Ru(Phen)_3_]^2+^-PLIM in the intact cell without disturbing the sensitivities of these molecular probes. The aim was also to demonstrate the stability of Ru(Phen)_3_]^2+^ as a molecular probe that can be used in vitro for oxygen detection in the presence of various stress factors mimicking the oxidative stress: other molecular sensors, H_2_O_2_, and the irradiation as a form of photodynamic therapy in cancer cells.

## 2. Results and Discussion

### 2.1. Finding the Limitations of [Ru(Phen)_3_]^2+^ Oxygen Sensitivity in CAM

**Figure 1:**
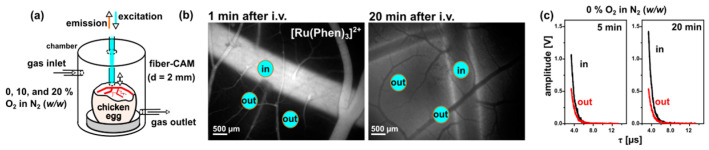
(**a**) Illustrative scheme of the gas chamber used to perform [Ru(Phen)_3_]^2+^ luminescence lifetime measurements in the CAM. (**b**) Fluorescence images of the CAM recorded 1 and 20 min after intravenous (iv) administration of [Ru(Phen)_3_]^2+^ (10 mg/kg of b.w.). (**c**) Illustrative outputs of [Ru(Phen)_3_]^2+^ (1 mg/kg of b.w.) luminescence lifetime detection at 0% O_2_ in N_2_ (*w*/*w*) atmosphere. The decays were detected in the blood vessels and out of the blood vessels as demonstrated in the fluorescence images.

The luminescence properties of [Ru(Phen)_3_]^2+^ were previously described [3,7,8,10,32]. As a small molecule, [Ru(Phen)_3_]^2+^ leaks out of the vessels and remains in the interstitial space of blood vessel cells [3]. Representative biodistribution of [Ru(Phen)_3_]^2+^ in the CAM at 1 and 20 min after its administration is presented in Figure 1b. While bright luminescence of [Ru(Phen)_3_]^2+^ was detected inside the vessels at 1 min post-administration, the majority of [Ru(Phen)_3_]^2+^ luminescence was observed out of the vessels at 20 min.

The luminescence decays of [Ru(Phen)_3_]^2+^ presented a typical monoexponential character (Figure 1c). Its lifetime rapidly changed with the absence of atmospheric oxygen. The [Ru(Phen)_3_]^2+^ luminescence lifetime sensitivity to oxygen in solutions is quite unambiguous [32]. Since the CAM is a living organism, it is more complex and heterogeneous than cells monolayers. The ideal mono-exponential decay observed in homogeneous solutions is, therefore, no longer observed in the CAM. This can be explained by the complexity and multi-component character of this living system. While the CAM membrane plexus, composed of endodermal cells, represents a static component, the dynamic blood flow is present in the whole system. 

Placement of the CAM into an atmosphere with low oxygen leads to significant changes in the oxygenation of the extracellular/interstitial space. However, the transport of oxygen at the cellular level and in the vessels is not a simple process. On the other hand, we had to keep the embryos alive during the measurements. For this reason, the chicken embryo kept residual oxygen supply as it can be recognized from Table 1.

Due to the non-Gaussian character of the [Ru(Phen)_3_]^2+^ luminescence lifetime (values detected in different CAMs) distributions, the data treatment could not be carried out by obvious reduction to arithmetic central values (mean values). Therefore, several central trend indicators were provided, as part of the R-programming environment [33]. The oxygenation values in Table 1 were estimated from Equation (1) (see below) that represents a calibration curve obtained from our previous study of [Ru(Phen)_3_]^2+^ in an isotonic solution of 0.9% NaCl [32]. The [Ru(Phen)_3_]^2+^ luminescence lifetimes measured at different regimes (0%, 10%, and 20% O_2_ in N_2_ for 5, 10, and 20 min) were converted to% oxygen by the Stern-Volmer relation as follows:

% O_2_ in N_2_ = (1/Mean *τ* − 919,155.47)/45,157.84. (1)

The ‘in’ values of oxygenation, which correspond to [Ru(Phen)_3_]^2+^ luminescence lifetimes inside the vessels, are higher than the ‘out’ values measured in the less vascularized areas. Moreover, the values detected during the first 5 min, which correspond to intravascular [Ru(Phen)_3_]^2+^, were also higher than at 10 and 20 min post-administration. We can see, that the % O_2_ in N_2_ (from 12 to 16) measured in the CAM at 20% and 10% O_2_ in N_2_ applied externally were not significantly different. At the contrary, 0% O_2_ in N_2_ regime (from 7 to 11) were significantly different from both 20 and 10% O_2_ in N_2_ regimes (*** *p* < 0.001).

**Figure 2:**
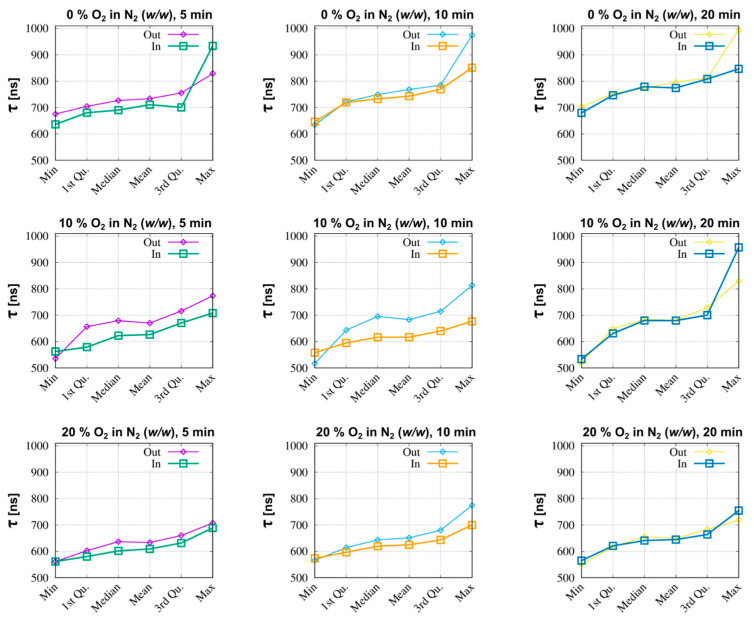
Statistical summaries of the [Ru(Phen)_3_]^2+^ luminescence lifetime measurements analysis in the CAM at 5, 10, and 20 min after iv administration and in atmospheres of 0%, 10%, and 20% O_2_ in N_2_ (*w*/*w*).

A statistical overview of the measurements collected under defined conditions is shown in Figure 2. This figure explicitly shows a structured six-dimensional summary(.) output of the program R [33], as previously described in the methods section. We can see that the use of this representation also reveals some equivalence with [Ru(Phen)_3_]^2+^ luminescence lifetime distribution characteristics. Initial findings revealed remarkable dynamic differences in statistics of ‘out’ and ‘in’ cases, as well as significant variations between results obtained with early, intermediate, and late administration. Let us first look at the values of the arithmetical means. These values can be arranged into the chronological layout of Table 1. Within this table, we specified the column where the generalized mean values of the [Ru(Phen)_3_]^2+^ luminescence lifetimes (τ) are transformed to the relative quantity.

rel_Md_ = 2(Median *τ* − Mean *τ*)/(Median *τ* + Mean *τ*). (2)

Here Median *τ* is a symbol for the median calculated from the data sets of luminescence decay times. More precisely, these are the values obtained by exponential regression. As a basis for comparison, we use the arithmetic mean of luminescence lifetime values denoted as Mean *τ*. For such averages, the zero of rel_Md_ indicates Gaussian behavior. From a practical point of view, obviously, it is important to know how the rel_Md_ differs from zero. An application example arose in the process of identifying 10% of O_2_ in N_2_ as the anomalous condition. Further, rel_Md_ (consider 0.029, 0.024, 0.03 to be representatively high values) becomes even more pronounced at 0% of O_2_ in N_2_.

For a more detailed interpretation, let’s also consider the ‘out-in’ sensitivity indicator:

rel_OI_ = 2(Mean_out_*τ* − Mean_in_*τ*)/(Mean_out_*τ* + Mean_in_*τ*). (3)

The indices ‘in’ and ‘out’ mean the selection of data under specific conditions. The relation is performed to conduct a statistical characterization of the corresponding ‘in’, ‘out’ pairs at given administration times under fixed O_2_% in N_2_ conditions. Table 1 shows a variation of a few percent with the outlier value of rel_OI_ = 0.103.

Hypoxic conditions applied to certain cells of the CAM may induce the fatal effect. This is of importance since the luminescence lifetime of [Ru(Phen)_3_]^2+^ in the presence of DNA is longer than in aqueous solution, and comparable with the values obtained in the absence of oxygen [10,11]. CAM cells suffering from acute hypoxia are more likely to become necrotic [34]. Cell membranes and nuclear envelopes of these cells can be more permeable for [Ru(Phen)_3_]^2+^ molecules. We hypothesize that this effect can explain the highest sensitivity to the oxygen level in the case of 10% O_2_ in N_2_ applied for 10 min to the CAM. Nevertheless, certain cells are not affected by these conditions. It is expected that more cells should suffer from hypoxia at the 0% O_2_ in N_2_ regimes. However, the [Ru(Phen)_3_]^2+^ luminescence lifetimes distribution is less dispersed. This identifiable anomaly is consistent with the results presented in Figure 2 which are highlighting the differences between the ‘in’ and ‘out’ curves that relate to the [Ru(Phen)_3_]^2+^ luminescence lifetime statistics.

Analysis of data based on multidimensional statistical inputs requires visual comparison and additional interpretation. The implementation of hierarchical processing in this project is new, but more natural because the clustering provides advanced tools to achieve interpretable and even visual consequence. In the present work, we also emphasize that clustering does not takes place once, but rather as an iterative mechanism with feedback.

**Figure 3:**
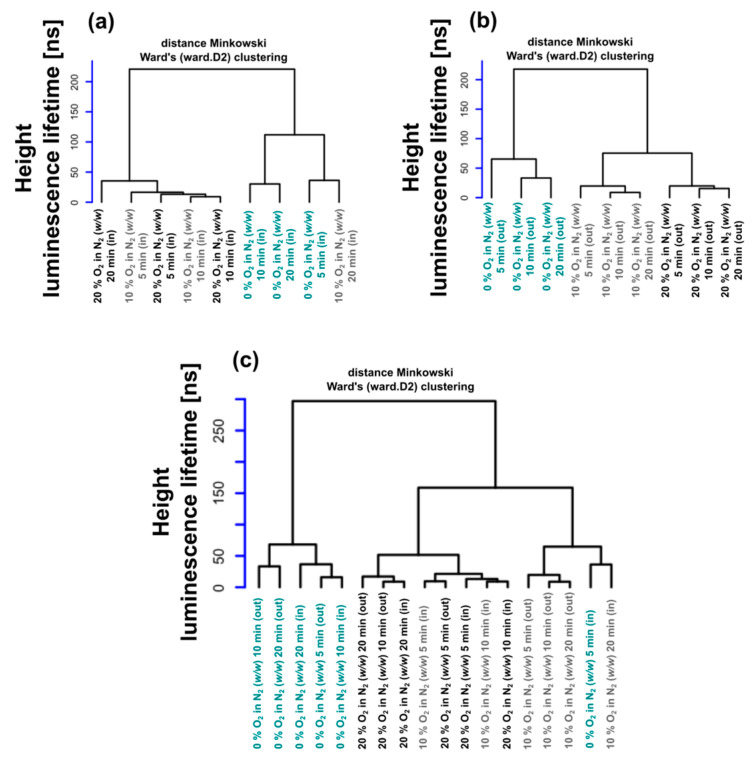
Dendrograms of [Ru(Phen)_3_]^2+^ luminescence lifetime detected (**a**) in the blood vessels and (**b**) out of the blood vessels of the CAM at 5, 10, and 20 min after iv administration and with O_2_ in N_2_ (*w*/*w*) atmosphere ranging between 0% and 20%. (**c**) The dendrogram, which is derived from the whole dataset showing mutual relationships.

Figure 3 shows the results of the cluster analysis based on luminescence lifetimes obtained under different experimental conditions. The specific arrangement of the results and conditions is such that, while (a) focuses on the ‘in’ cases, panel (b) monitors the ‘out’ scenarios. The color and the corresponding contents reflect the differences between panels (a) and (b). In panel (a) the structure of the dendrogram is more fragmented, while (b) is more ordered. This can be explained by the experimental control (high tissue homogeneity) of the ‘out’ cases, whereas the ‘in’ case data also reflect more complex physiological internal processes.

The results obtained with 10% O_2_ in N_2_ are the most fragmented, as shown in the overall statistics in panel (c). This is in agreement with the aforementioned anomaly analysis expressed by means of the rel_OI_ indicator. The cophenetic distance in the case of the panel (c) also clearly shows that while 10% and 20% O_2_ in N_2_ are statistically close ([Ru(Phen)_3_]^2+^ luminescence lifetime height 150 ns, highest square arch), the case of 0% O_2_ in N_2_ dataset cases is a significant outlier ([Ru(Phen)_3_]^2+^ luminescence lifetime height = 270 ns).

**Figure 4:**
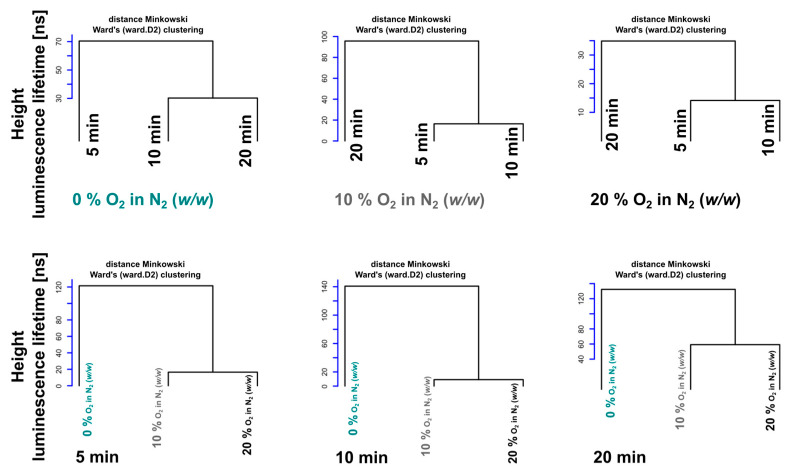
System of sub-dendrograms obtained from [Ru(Phen)_3_]^2+^ luminescence lifetime datasets detected in the blood vessels of the CAM at 5, 10, and 20 min after i.v. administration and 0–20% O_2_ in N_2_ (*w*/*w*) atmosphere. The lifetime data set visualization is divided into an upper and a lower part. Each part consists of three dendrograms, which are composed of separately processed data (time-dependent, O_2_ in N_2_ concentration-dependent). While the first three dendrograms are constructed according to the similarity of the data at given concentrations, the other three are constructed at given detection times.

However, one might ask where the roots of mismatch and fragmentation are within the ‘in’ cases. The answer is indicated by the three upper panels of Figure 4, which were generated by the separate processing of (‘in’, 0%), (‘in’, 10%), and (‘in’, 20%) subsets grouped from the entire ‘in’ statistics. The administration time (read the dendrogram from left to right) of the legs (branches) changed significantly from the 0% sequence (5 min, 10 min, and 20 min) to (20 min, 5 min, and 10 min) in the cases of 10% and 20% O_2_ in N_2_. This means that conditions corresponding to the sensitive branch with a duration of 20 min behave as if it more closely mimics the distance conditions of ‘in’ case. Another perspective of data perception is established in the last three ‘in’ dendrograms where the selection of data for clustering is carried out according to the time after administration. Maintaining the canonical order (0%, 10%, and 20%) is a surprising result of these experiments. Here, too, an unusual dendrogram height shift occurs at 20 min.

We recommend reading Figure 3 panels in two directions. One can proceed from the details of the panels (a) and (b), and connect them in the direction of the composite panel (c) as the results. On the other hand, there is a top-down, reductionist way of interpreting panel (c) with less detailed visual representations of (a) and (b). Figure 4 provides alternative and complementary methods for processing and understanding the results.

We know from previous examples as well as from practical experience that because of their flexibility, straightforwardness, and controllability, the dendrogram and cluster methods could still compete with many other forms of machine learning and data processing. They have become known and used not only in population genetics but also in many other bio-motivated fields, including brain sciences [35].

We assumed that the change of oxygen atmosphere from normoxia to hypoxia in the gas chamber may have induced oxidative stress. The level of oxidative stress in CAM cells may then locally influence [Ru(Phen)_3_]^2+^ luminescence lifetime detection. 

In the next step, we have evaluated the influence of the oxidative stress on the [Ru(Phen)_3_]^2+^ luminescence lifetime detection in glioma cells.

### 2.2. Localization of [Ru(Phen)_3_]^2+^ in Glioma Cells and Oxidative Stress

**Figure 5:**
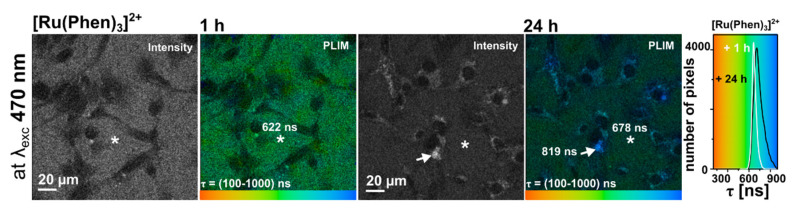
Four images on the left: Illustrative intensity and PLIM images obtained with 200 µM [Ru(Phen)_3_]^2+^ applied in the culture medium of U87 MG cells for 1 and 24 h. Right: [Ru(Phen)_3_]^2+^ luminescence lifetime distribution histograms (1 h—white, 24 h black). The luminescence lifetimes are color-coded (100 ns—red, 1000 ns—blue). The samples were excited with laser light at 470 nm. The white arrows point to intracellular and asterisks to extracellular localization of [Ru(Phen)_3_]^2+^.

We have used a unique FLIM/PLIM system to see how [Ru(Phen)_3_]^2+^ localization influences the [Ru(Phen)_3_]^2+^ luminescence lifetime. Illustrative PLIM images of [Ru(Phen)_3_]^2+^ in the extracellular and intracellular spaces are presented in Figure 5. U87 MG cells incubated during 1 h with 200 µM [Ru(Phen)_3_]^2+^ are recognized as the dark areas in the intensity and PLIM images. Extracellular [Ru(Phen)_3_]^2+^ luminescence lifetime was ≈650 ns (according to Equation (3) it corresponds to 13.7% of O_2_ in N_2_). [Ru(Phen)_3_]^2+^ was not recognized in the cytoplasm. However, the subcellular vesicles loaded with [Ru(Phen)_3_]^2+^ were identified at longer (24 h) incubations time. We have previously demonstrated that [Ru(Phen)_3_]^2+^ crossed the plasma membrane via endocytosis and maintained localized in the endocytotic vesicles, and peroxisomes [7]. The intracellular lifetimes of [Ru(Phen)_3_]^2+^ were longer (≈800 ns and 7.3% of O_2_ in N_2_) than for extracellularly measured. Global lifetimes, i.e., the extracellular plus intracellular components, as represented by luminescence lifetime distribution histograms (Figure 5), fall in the interval ranging between 600 and 900 ns (16.5% and 4.2% of O_2_ in N_2_). The increase of the intracellularly localized [Ru(Phen)_3_]^2+^ luminescence lifetime reflects lower oxygenation within the cells. However, the luminescence lifetime also may dependents on other environmental factors that could explain the longer luminescence lifetimes.

**Figure 6:**
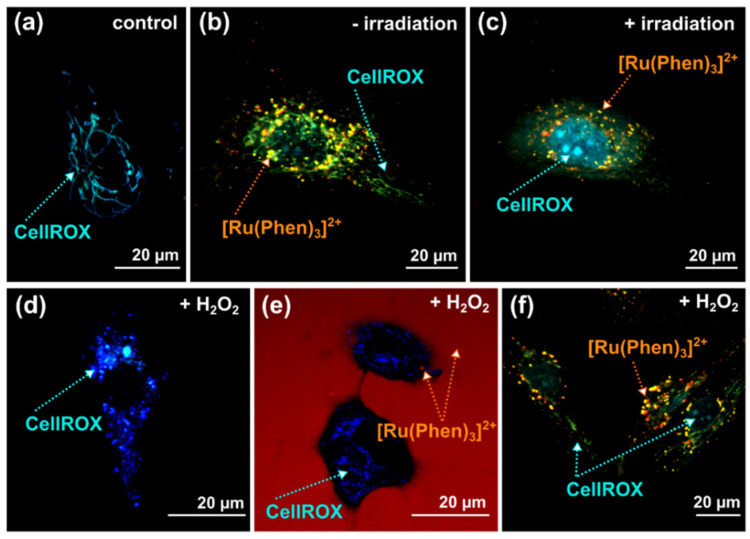
Illustrative confocal microscopy images of U87 MG cells stained with (**a**–**f**) 1 µM (30 min) CellROX^®^Green (cyan and blue) and 200 µM [Ru(Phen)_3_]^2+^ (orange and red). These dyes were applied in the U87 MG cells culture medium for (**e**) 1 and (**b**,**c**,**f**) 24 h. (**c**) The cells were irradiated with microscopy (detection time less than 2 min, 2% of the power of the laser: 405 and 488 nm)—the second scan of cells stained with [Ru(Phen)_3_]^2+^ and CellROX^®^Green. The photoreaction induced oxidative stress. (**d**–**f**) Extracellular oxidative stress was induced by 200 µM H_2_O_2_.

[Ru(Phen)_3_]^2+^ can be considered as a metalotoxin. However, it has a low toxicity and phototoxicity in comparison with porphyrins, as we have already demonstrated [3,7,8]. The presence of metalotoxin in cells may induce oxidative stress. One of the most sensitive organelles to oxidative stress in cells are mitochondria. Representative confocal fluorescence images of CellROX green, a probe sensitive to oxidative stress induction in mitochondria, are presented in Figure 6. We have selected this ROS unspecific probe due to its spectral properties and subcellular localization which are different from those of [Ru(Phen)_3_]^2+^ (Figure 6b). Comparison of CellROX green localization with rhodamine 123 (a mitochondrial probe sensitive to mitochondria membrane potential), and [Ru(Phen)_3_]^2+^ is presented in Figure S1 in Supplementary Materials. Intact mitochondria in U87 MG cells were detected in the presence of [Ru(Phen)_3_]^2+^. Application of light with the microscopy detection system-induced photodamages in cells, as revealed by the destabilization of the mitochondria membrane potential (tubular structures cannot be recognized, see Figure S2 in Supplementary Materials) and an increase in oxidative stress (CellROX localization in the nucleus), as can be seen in Figure 6c. The localization of [Ru(Phen)_3_]^2+^ before and after illumination did not change (Figure 6b,c).

To see the stability of [Ru(Phen)_3_]^2+^ distribution and luminescence at external induction of oxidative stress, we have applied H_2_O_2_ into the cell culture. The presence of 200 µM H_2_O_2_ in U87 MG resulted in significant reduction of intracellular catalase and superoxide dismutase 1 (see Figure S3 in the Supplementary Materials). Zhang et al. observed that apoptosis triggered by H_2_O_2_ mediated oxidative stress in hepatoma cells involved decreasing of catalase and superoxide dismutase activity [36]. It is in agreement with our findings. In our study, we have applied H_2_O_2_ for short time (<1 h) to induce acute stress, and prevent its complete metabolism. We have previously demonstrated by MitoSOX^TM^ Red [15] that superoxide production increased in U87 MG cells treated with H_2_O_2_. Besides, application of H_2_O_2_ resulted in significant ROS production extracellularly and intracellularly detected by DCFDA/H2DCFDA assay (see Figure S4 in Supplementary Materials), and further caused significant lipid peroxidation inside the cells (see Figure S5 in Supplementary Materials).

The extracellular application of H_2_O_2_ (without molecular probes) induced dissipation of mitochondrial membrane potential, and fission of mitochondria in U87 MG cells (Figure 6d and Figure S2). The same effect as in the absence of [Ru(Phen)_3_]^2+^ (fission of mitochondria) was observed on mitochondria in the presence of [Ru(Phen)_3_]^2+^ and H_2_O_2_ keeping in the cell culture medium (Figure 6e). [Ru(Phen)_3_]^2+^ distribution and luminescence were not changed.

A similar experiment was performed in cells that were incubated for 24 h with [Ru(Phen)_3_]^2+^, at that time it crossed plasma membrane via endocytosis [7]. In this case, [Ru(Phen)_3_]^2+^ was removed from the cell culture medium prior observation. The expected fission of mitochondria induced by H_2_O_2_ was less effective (Figure 6f). Indeed, oxidative stress induced by H_2_O_2_ caused partial relocalization of CellROX into the nucleus. The number of [Ru(Phen)_3_]^2+^ loaded vesicular structures previously identified as the peroxisomes [7] were localized nearby tubular structured mitochondria (see Figures S1 and S6 in Supplementary Materials), where it may attenuate the oxidative stress. This finding strongly suggests the antioxidants-like effects of [Ru(Phen)_3_]^2+^. The partial localization of CellROX in the nuclei, on the other hand, suggests that oxidative stress level increased in these cells due to H_2_O_2_.

### 2.3. [Ru(Phen)_3_]^2+^ Luminescence Lifetime Stability during Oxidative Stress in Solution

**Figure 7:**
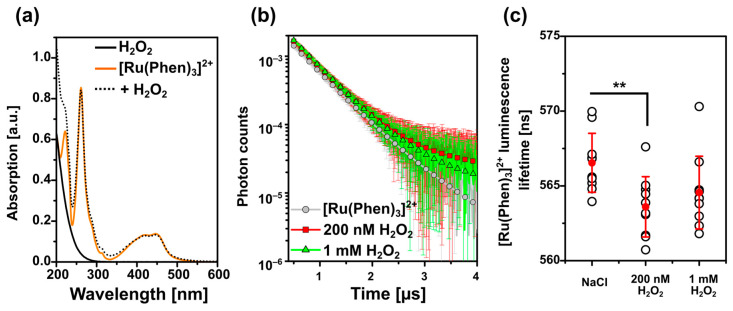
(**a**) Absorption spectra of 1 mM H_2_O_2_ (black), 10 µM [Ru(Phen)_3_]^2+^ (orange), and [Ru(Phen)_3_]^2+^ with 1 mM H_2_O_2_ (dotted black) 0.9% NaCl solutions. (**b**) 10 µM [Ru(Phen)_3_]^2+^ luminescence lifetime decays in 0.9% NaCl physiological solution (grey circles) and the presence of 200 nM (red squares) and 1 mM H_2_O_2_ (green triangles). (**c**) [Ru(Phen)_3_]^2+^ luminescence lifetime values detected at the conditions mentioned in (**b**). The mean values of the decays and the lifetimes are the averages of ten measurements with the appropriated standard deviations as the error bars. The level of significance was assessed with the Student *t*-test: ** *p* < 0.01.

We have demonstrated that the localization of [Ru(Phen)_3_]^2+^ in cells was the same in the presence and absence of H_2_O_2_. We assumed that the [Ru(Phen)_3_]^2+^ luminescence lifetime was more sensitive to this molecule than its intensity. For this reason, we have conducted [Ru(Phen)_3_]^2+^ luminescence lifetime measurements in the solutions of H_2_O_2_. Figure 7a demonstrates unaffected absorption spectra of [Ru(Phen)_3_]^2+^ in the presence and absence of H_2_O_2_. The decays of [Ru(Phen)_3_]^2+^ luminescence lifetimes are plotted in Figure 7b. The extracted values of [Ru(Phen)_3_]^2+^ lifetimes were found to be significantly different in the presence of low concentrated (200 nM) H_2_O_2_ (Figure 7c). A small decrease of the [Ru(Phen)_3_]^2+^ lifetimes could be connected with the decomposition of H_2_O_2_ with time. The oxygen developed in this reaction can induce quenching of the [Ru(Phen)_3_]^2+^ luminescence lifetime. The highest concentration of H_2_O_2_ did not induce further shortening of the lifetimes. The deviations of the luminescence lifetime values induced by H_2_O_2_ were small, i.e., within the range (560–575 ns). Estimated values of applied O_2_ in N_2_ (19–18%) were higher in solutions in comparison to the % of O_2_ in N_2_ detected in the CAM. This suggests that the effect of H_2_O_2_ on the [Ru(Phen)_3_]^2+^ luminescence lifetime is minor.

### 2.4. MTO Fluorescence and [Ru(Phen)_3_]^2+^ Luminescence Lifetimes Sensitivity during Oxidative Stress Induced in Glioma Cells

In our previous study, we have demonstrated that oxidative stress induced in cancer cells fragmented MTO such a way that one fraction was bound to DNA in the nucleus resulting in an increase of MTO fluorescence lifetime [15]. Interestingly, MTO fluorescence lifetime increased in mitochondria in which oxidative stress level increased. For this reason, we have selected MTO to detect oxidative stress levels and [Ru(Phen)_3_]^2+^ to detect oxygenation in cells with a unique time-resolved microscopy system. With this setup, excitation and emission of MTO and [Ru(Phen)_3_]^2+^ were clearly discriminated, i.e., no signals of MTO was detected in PLIM, whereas no [Ru(Phen)_3_]^2+^ luminescence was detected in the MTO FLIM channel.

**Figure 8:**
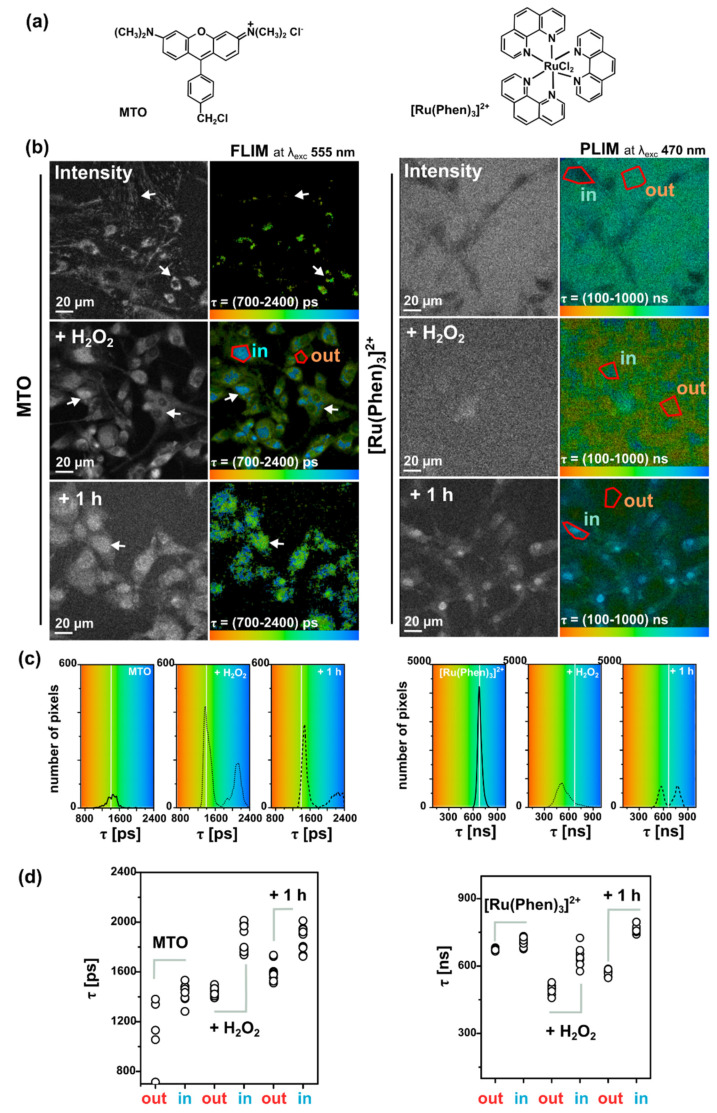
(**a**) Chemical structures of MTO (left) and [Ru(Phen)_3_]^2+^ (right). (**b**) Representative FLIM and PLIM images of MTO (left) and [Ru(Phen)_3_]^2+^ (right) individually applied in the medium of U87 MG cells for 30 min in the absence and presence of 200 µM H_2_O_2_ (shortly and 1 h after H_2_O_2_ administration). White arrows point to MTO mitochondrial and nuclear localization. The MTO fluorescence lifetimes ROI was selected in the perinuclear/nuclear area (in) and far from this area (out). The [Ru(Phen)_3_]^2+^ luminescence lifetimes ROI were selected in the intracellular (in) and extracellular area (out). (**c**) MTO and [Ru(Phen)_3_]^2+^ luminescence lifetime distribution histograms. The luminescence lifetimes are color-coded (minima—red, maxima—blue). Gray-scaled images represent the luminescence intensity of MTO and [Ru(Phen)_3_]^2+^. The samples were excited with laser light at 555 nm (MTO) and 470 nm ([Ru(Phen)_3_]^2+^). (**d**) MTO and [Ru(Phen)_3_]^2+^ luminescence lifetime values detected at the conditions mentioned in (**b**). At least ten representative ROI was selected.

Oxidative stress was induced externally by H_2_O_2_. Figure 8 shows FLIM images of MTO fluorescence lifetimes and PLIM images of [Ru(Phen)_3_]^2+^ luminescence lifetimes in U87MG cells in the presence and absence of H_2_O_2_. We can recognize the increase of MTO fluorescence lifetime (blue-colored) in the presence of H_2_O_2_ (Figure 8a). Two main regions of interest (ROI) in cells were recognized. One ROI is in the perinuclear area (in, ≈1500 ps) with long MTO fluorescence lifetimes, whereas the second ROI is in the periphery, nearby the plasma membrane (out, ≈1200 ps) with shorter MTO fluorescence lifetimes (Figure 8b,c). As the intracellular H_2_O_2_ concentration increased, the MTO delocalizes into the nucleus and the MTO fluorescence lifetime dramatically increased in this region (≈1900 ps).

PLIM measurements of [Ru(Phen)_3_]^2+^ were performed within 1 h after its administration, i.e., when [Ru(Phen)_3_]^2+^ localized extracellularly. Dark areas in images showing the cell localization of [Ru(Phen)_3_]^2+^ in PLIM can be recognized in Figure 8a. Application of H_2_O_2_ resulted in a decrease of the extracellular [Ru(Phen)_3_]^2+^ luminescence lifetimes from ≈650 ns (13.7% of O_2_ in N_2_) to ≈400 ns (35% of O_2_ in N_2_), as presented in Figure 8b. As observed in solutions, this effect could be caused by the H_2_O_2_ decomposition. The PLIM detection at longer incubation time (1 h) after the H_2_O_2_ application developed a bimodal character of [Ru(Phen)_3_]^2+^ luminescence lifetimes (see the distribution histograms in Figure 8c). The shorter lifetimes < 600 ns (>16.5% of O_2_ in N_2_) were detected in the extracellular area, whereas the longer lifetime > 600 ns (<16.5% of O_2_ in N_2_) were detected in cells. It should be noted that the nuclei of the cells were brightly labeled with [Ru(Phen)_3_]^2+^ and with the longest luminescence lifetimes. This can be explained by the interaction of [Ru(Phen)_3_]^2+^ with cellular DNA. Indeed, Komor et al. described that the luminescence of [Ru(Phen)_3_]^2+^ increased in the presence of DNA [10,11]. We assume that the H_2_O_2_ applied destabilized the plasma membrane, which became permeable to [Ru(Phen)_3_]^2+^ molecules. These results suggest that the evaluation of [Ru(Phen)_3_]^2+^ lifetimes and subsequent determination of oxygenation level strongly depend on cell fitness.

**Figure 9:**
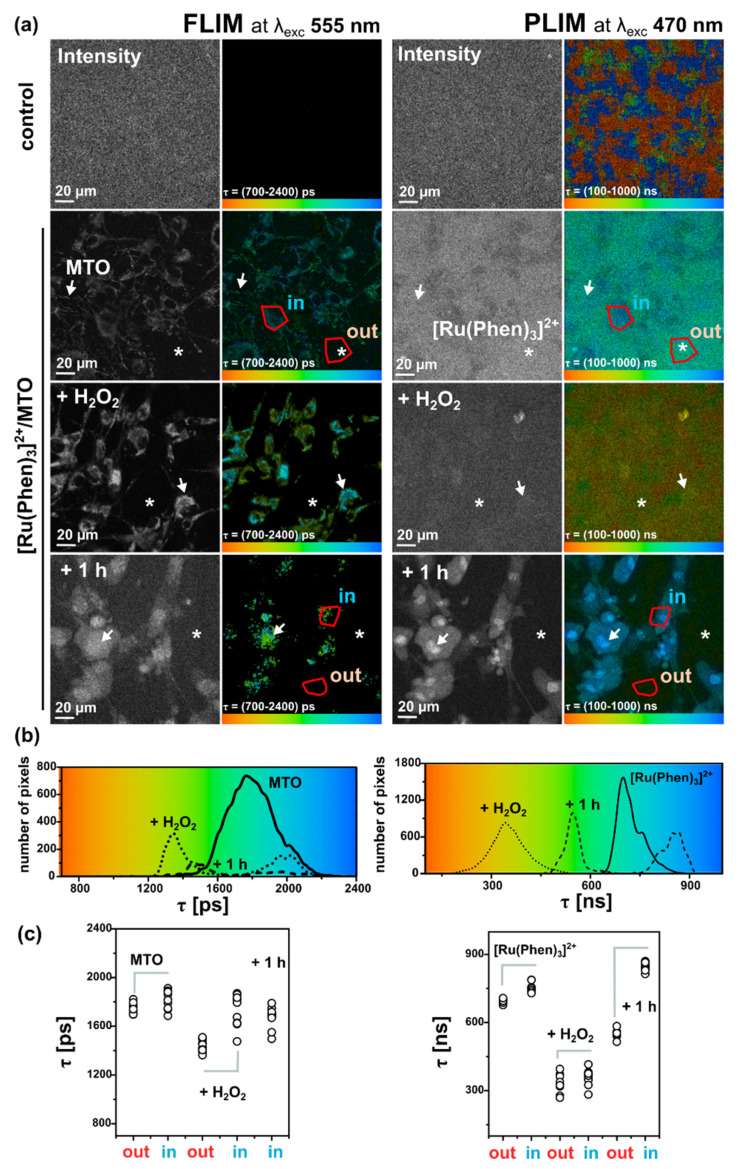
(**a**) Representative FLIM and PLIM images of MTO (left) and [Ru(Phen)_3_]^2+^ (right) both applied in the medium of U87 MG cells for 30 min in the absence and presence of 200 µM H_2_O_2_ (shortly and 1 h after H_2_O_2_ administration). The cells without staining were collected in the FLIM and PLIM channels as well. White arrows point to MTO mitochondrial and nuclear localization. The MTO fluorescence lifetimes ROI was selected in the perinuclear/nuclear area (in) and far from this area (out). The [Ru(Phen)_3_]^2+^ luminescence lifetimes ROI were selected in the intracellular (in) and extracellular (white asterisk) area (out). (**b**) MTO and [Ru(Phen)_3_]^2+^ luminescence lifetime distribution histograms. The luminescence lifetimes are color-coded (minima—red, maxima—blue). Gray-scaled images represent the luminescence intensity of MTO and [Ru(Phen)_3_]^2+^. The samples were excited with laser light at 555 nm (MTO) and 470 nm ([Ru(Phen)_3_]^2+^). (**c**) MTO and [Ru(Phen)_3_]^2+^ luminescence lifetimes detected at the conditions mentioned in (**a**). At least ten representative ROIs were selected.

One original aspect of our work was to apply simultaneously MTO and [Ru(Phen)_3_]^2+^ in cell culture media and to record FLIM and PLIM images from the same cells to monitor oxidative stress level and oxygenation changes. MTO FLIM and [Ru(Phen)_3_]^2+^ PLIM images were detected in the absence and presence of H_2_O_2_. No detectable FLIM and PLIM signals were observed in U87 MG cells in the absence of MTO and [Ru(Phen)_3_]^2+^ (Figure 9a). As can be seen in Figure 9, we have obtained results comparable to those observed when MTO and [Ru(Phen)_3_]^2+^ were applied independently. The MTO fluorescence lifetimes increased and the nucleus localized MTO appeared after H_2_O_2_ application. The [Ru(Phen)_3_]^2+^ luminescence lifetimes decreased shortly after H_2_O_2_ application and increased with time and nuclear distribution (Figure 9b,c). It should be noted that the oxidative stress level evidenced by the MTO fluorescence lifetime increased in the presence of [Ru(Phen)_3_]^2+^ in the perinuclear area. Besides, the [Ru(Phen)_3_]^2+^ luminescence lifetime slightly increased in the presence of MTO. Therefore, the mutual influence of [Ru(Phen)_3_]^2+^ and MTO should be taken into consideration when oxidative stress level and oxygenation are estimated simultaneously.

### 2.5. MTO Fluorescence and [Ru(Phen)_3_]^2+^ Luminescence Lifetimes Changes Observed during PDT Induced by Curcumin and Blue Light in Glioma Cells

In the following, we aimed to apply the simultaneous FLIM/PLIM detection of MTO and [Ru(Phen)_3_]^2+^ after intracellular induction of oxidative stress by PDT. We have tested the hypothesis to detect oxygen consumption in the extracellular medium (with [Ru(Phen)_3_]^2+^), and oxidative stress (with MTO) induction within the cells by PDT.

The absorption curves of [Ru(Phen)_3_]^2+^, MTO, and curcumin are partially overlapping. Therefore, intensity-based techniques (e.g., fluorescence microscopy) present obstacles to discriminate their emissions. In our laboratory, we utilized a time-resolved microscopy system that enabled us to detect fluorescence (FLIM) and phosphorescence (PLIM) lifetimes from sample (cell) after its excitation with a white femtosecond laser. Thanks to this system we could differentiate by time-resolved spectroscopy the [Ru(Phen)_3_]^2+^, MTO, and curcumin localization in the same cell. Moreover, the lifetimes of MTO-FLIM and [Ru(Phen)_3_]^2+^ provided information regarding the oxidative stress level and oxygenation of the sample [3,15].

As mentioned in the introduction, curcumin can be a scavenger of singlet oxygen but, under irradiation with blue light, it can produce superoxide radicals, singlet oxygen and other reactive oxygen species [29,30,31]. A combination of dual-detection (MTO-[Ru(Phen)_3_]^2+^) could reveal both, oxidative stress and antioxidant effect of curcumin during PDT in the same cells. 

**Figure 10:**
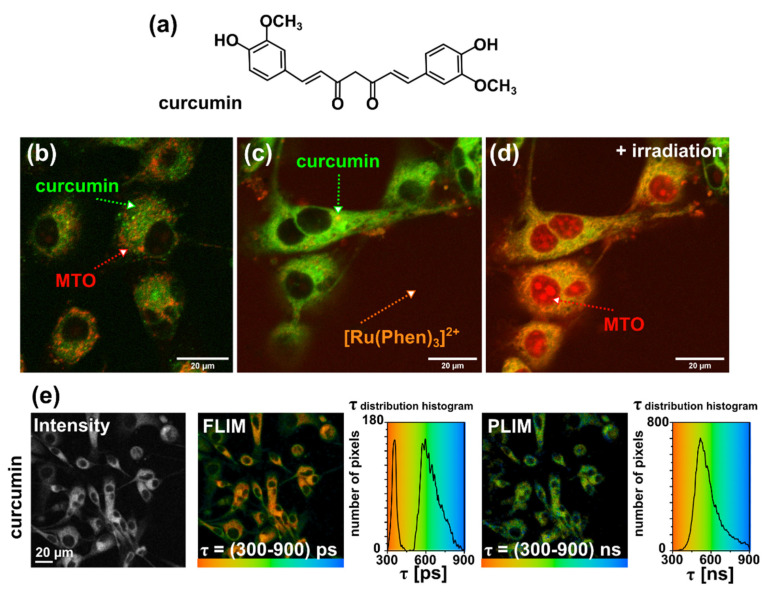
(**a**) Chemical structure of curcumin. Illustrative confocal microscopy fluorescence intensity images of U87 MG cells stained with (**b**) 10 µM curcumin (green) and 400 nM MTO (red), (**c**) curcumin, MTO (localized in the mitochondria), and 200 µM [Ru(Phen)_3_]^2+^ (red, extracellularly localized) first scan and (**d**) second scan that induced oxidative stress (MTO is localized in the nuclei). (**e**) FLIM and PLIM images of curcumin in U87 MG cells and corresponding fluorescence and phosphorescence lifetimes histograms. The lifetimes are color-coded (minima—red, maxima—blue). The gray-scaled image represents the fluorescence and phosphorescence intensity of curcumin.

In the present study, we have incubated U87 MG cells with 10 µM curcumin during 1 h. Representative fluorescence intensity images of curcumin, MTO, and [Ru(Phen)_3_]^2+^ are presented in Figure 10. We have observed an intracellular localization of curcumin, whereas MTO localizes in mitochondria, and [Ru(Phen)_3_]^2+^ in the extracellular space. Irradiation (detection time less than 3 min, laser was set at 2% of the total power of the laser: 405, 488, and 555 nm) coming from the microscopy system during fluorescence imaging triggered photoreactions generated oxidative stress production, photobleaching of curcumin fluorescence, and nuclear localization of MTO (Figure 10c). The intracellular localization of curcumin was not specifically in mitochondria. This is in agreement with the observations performed by Sala de Oyanguren et al. who demonstrated specific localization of curcumin in the endoplasmic reticulum and described its role in the process of autophagy and apoptosis [37].

The fluorescence lifetime of curcumin is short (≈hundreds of ps), as reported by Khopde et al. in different solvent [38]. FLIM and PLIM images of curcumin were detected in the same spectral window as [Ru(Phen)_3_]^2+^. Fluorescence lifetime histograms of curcumin have a bimodal character (Figure 10e). Short components (≈350 ps) were localized in the perinuclear area, whereas longer fluorescence lifetimes of curcumin (≈580 ps) were observed in the periphery near the plasma membrane. In comparison to fluorescence, very little is known about curcumin phosphorescence. In the nineties, Chignell et al. described the photobleaching of curcumin and reported that its phosphorescence depends on the excitation wavelength [29]. A broad phosphorescence spectrum > 650 nm was observed at 77 K and 282 nm excitation by this group. In the present study, the phosphorescence lifetime of curcumin was observed in the same PLIM detection window as it was for [Ru(Phen)_3_]^2+^ (Figure 10d). Phosphorescence lifetimes of ≈590 ns were detected within U87 MG cells. To the best of our knowledge, this is the first time that curcumin phosphorescence imaging is demonstrated. We should note, that possible other product of curcumin degradation in the cytoplasm could be detected in PLIM mode. This effect should be verified in the future study. Very often the PS is degraded during PDT due to oxidative stress [39]. For this reason, the curcumin PLIM, in the present study, may be considered as a marker for PDT efficacy in cells. With this regard, curcumin degradation should result in decrease of its phosphorescence.

**Figure 11:**
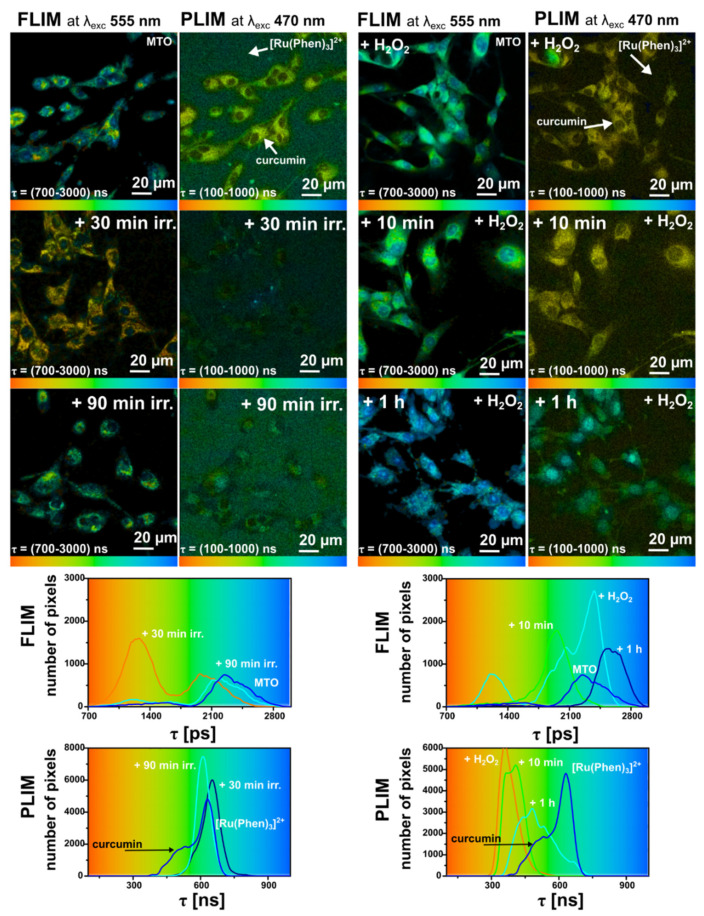
Illustrative FLIM images of MTO and PLIM images of [Ru(Phen)_3_]^2+^ both applied in the medium of U87 MG cells for 30 min in the presence of 10 µM curcumin. The phosphorescence of curcumin (localized only in cells) was detected in the same channel as [Ru(Phen)_3_]^2+^. The cells were irradiated 30 and 90 min with blue light, 463 ± 10 nm with an irradiance of 100 µW/cm^2^ (six images in the upper left). Alternatively, the cells were stressed (six images in the upper right) with 200 µM H_2_O_2_ (10 min and 1 h after H_2_O_2_ administration). FLIM and PLIM luminescence lifetime histograms are plotted for both conditions (below the images). The luminescence lifetimes are color-coded (minima—red, maxima—blue).

In the following, curcumin was irradiated in cells with blue light emitted by a diode (463 ± 10 nm and 100 µW/cm^2^ irradiance) for 30 and 90 min. Our time-resolved microscopy system detected the FLIM image of MTO and PLIM images of curcumin and [Ru(Phen)_3_]^2+^ in the same cell. Irradiation of cells in the presence of [Ru(Phen)_3_]^2+^ only and in combination with MTO in the absence of curcumin did not result in significantly different changes in FLIM/PLIM images (see Figures S7 and S8 in Supplementary Materials). Indeed, irradiation of cells in the presence of [Ru(Phen)_3_]^2+^ resulted in significant ROS formation, and detected with H2DCFDA sensor in the extracellular area (see Figure S4 in Supplementary Materials). It should be noted that higher light dose may induce certain damages as it was observed by confocal fluorescence microscopy. However, the presence of MTO during irradiation resulted to photodamage of the cells that affected FLIM/PLIM detection. The FLIM and PLIM images of MTO, curcumin, and [Ru(Phen)_3_]^2+^ are presented in Figure 11. We can see that the presence of curcumin in U87 MG cells without irradiation increases the MTO fluorescence lifetimes (>2000 ps). This suggests that curcumin increases the oxidative stress in cells. The luminescence lifetimes of [Ru(Phen)_3_]^2+^ were not modified by the presence of curcumin (≈700 ns; 11.28% of O_2_ in N_2_). The histograms of curcumin phosphorescence lifetimes revealed values as short as ≈400 ns.

Irradiation dramatically changed the MTO and curcumin luminescence lifetime images. Indeed, the MTO fluorescence lifetimes decreased below 1400 ps after 30 min irradiation with blue light. Longer irradiation induced an increase of MTO fluorescence lifetimes. This suggests that the oxidative stress firstly decreased and later increased in cells along the irradiation time. We can recognize that the phosphorescence of curcumin was quenched by the 30 min irradiation with blue light, which led to a slight increase of the [Ru(Phen)_3_]^2+^ luminescence lifetime from 631 to 658 ns (from 14.7% to 13.3% of O_2_ in N_2_). These changes can be seen in the histograms of [Ru(Phen)_3_]^2+^ and curcumin phosphorescence lifetimes (Figure 11). It could be that these changes are due to a slight oxygen consumption. On the contrary, the longest 90 min irradiation resulted in decreasing [Ru(Phen)_3_]^2+^ luminescence lifetimes down to 613 ns (15.7% of O_2_ in N_2_). It should be noted that the irradiation of cells during FLIM/PLIM detection did not induce any photodamage as it was the case for the confocal fluorescence microscopy.

One can reasonably assume that the antioxidant effects of irradiated curcumin prevail for short irradiation times. The photobleaching of curcumin probably induced oxygen consumption ([Ru(Phen)_3_]^2+^ luminescence lifetimes increasing). Moreover, degraded curcumin fractions, active as antioxidants, reduced the oxidative stress level in cells (MTO fluorescence lifetime decreasing).

Long-time irradiation of U87 MG cells probably reversed the curcumin antioxidant effect to photodamage induction. The oxidative stress level (MTO fluorescence lifetimes) increased in those cells. The cells became leakier for [Ru(Phen)_3_]^2+^ molecules, which were internalized in the cells. However, we could not distinguish between the signal from [Ru(Phen)_3_]^2+^ and possible photoproducts of curcumin that may remain in cells after irradiation and create bias in the [Ru(Phen)_3_]^2+^ luminescence lifetime determination.

As we have demonstrated above (Section 2.2), extracellular H_2_O_2_ induced significant oxidative stress in cells. In one of our previous study, we have demonstrated that this effect can be reduced by catalase (antioxidant) application [15]. One can assume that curcumin-PDT may induce H_2_O_2_ production in cells and subsequently increase intracellular catalase concentration. For this reason, the oxidative stress was induced extracellularly by H_2_O_2_ in U87 MG cells treated with curcumin (Figure 11). Indeed, we have first observed a decrease of oxidative stress (decreased MTO fluorescence lifetimes), and a decrease of [Ru(Phen)_3_]^2+^ luminescence lifetimes. This is consistent with the results obtained in the absence of curcumin (Figure 8 and Figure 9). Interestingly, short phosphorescence lifetimes of curcumin were detected in cells. As expected, an increase of MTO fluorescence and [Ru(Phen)_3_]^2+^ luminescence lifetimes were observed 1 h after H_2_O_2_ administration. These results suggest that the H_2_O_2_ concentration was too high to be reduced by curcumin.

Although we have demonstrated that curcumin possesses antioxidant properties this compound is not as antioxidating as catalase. On the other hand, curcumin can be degraded with H_2_O_2_. The resulting products of this reaction could result in their interaction with curcumin, and decrease its phosphorescence lifetime. Low concentrations of curcumin at 1 h after H_2_O_2_ administration disable the antioxidant effect of curcumin and could be another reason for higher for the oxidative stress. 

Photodamages induced in cells by curcumin differ from massive injury caused by H_2_O_2_. For this reason, we have conducted an assessment of the metabolic activity and phototoxicity in U87 MG cells in the presence of all studied molecules before and after irradiation with the blue light. Those results also define the limits of simultaneous MTO and [Ru(Phen)_3_]^2+^ luminescence lifetimes detection with minimal phototoxicity. 

**Figure 12:**
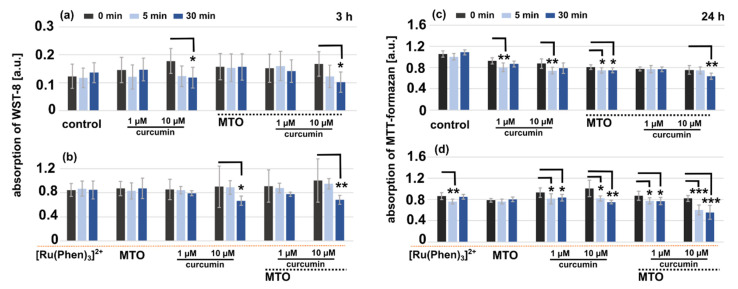
Metabolic activity test of U87 MG cells performed with (**a**,**b**) WST-8 and (**c**,**d**) MTT-formazan 3 and 24 h after the irradiation, respectively. The cells were pre-treated during 1 h with 1 and 10 µM curcumin, 400 nM MTO, and 200 µM [Ru(Phen)_3_]^2+^, individually or in combinations, as represented by histograms. The histograms present the results obtained in the (**a**,**c**) absence, and (**b**,**d**) presence of [Ru(Phen)_3_]^2+^. The cells were irradiated with blue light (463 ± 10 nm; 100 µW/cm^2^) for 5 and 30 min. The average values presented correspond to the means of two independent measurements. Error bars: standard deviations from the mean values. The level of significance was estimated by the Student *t*-test: * *p* < 0.05, ** *p* < 0.01, and *** *p* < 0.001.

Two irradiation times were applied: 5 min and 30 min. We have chosen to irradiate cells for 5 min to evaluate if the irradiation during PLIM/FLIM measurement may induce photodamages in the cells. The evaluation was performed in living cells 3 h after irradiation with the WST-8 kit (Figure 12a,b). This kit is biocompatible for living cells and does not requires DMSO administration. Standard MTT-assays were performed 24 h after cell irradiations (Figure 12c,d). It should be noted, that the absorption of WST-8 overlaps with that of [Ru(Phen)_3_]^2+^, MTO, and curcumin. For this reason, a level of significance was estimated within the studied sets. A significant decrease of metabolic activity was found 30 min and 3 h after the irradiations in U87 MG cells treated with 10 µM curcumin, and in the presence of MTO (Figure 12a). The metabolic activity of those cells was remarkably suppressed 24 h after the irradiation (Figure 12c). Presence of [Ru(Phen)_3_]^2+^ in cells amplified the photoeffect (Figure 12b,d). However, the viability of these cells did not drop below 60%. These results suggest that the antioxidizing activity of curcumin is more important than the apoptotic one. Our results suggest that a simultaneous detection of [Ru(Phen)_3_]^2+^ and MTO can be performed without photodamages if the irradiation from excitation laser maintain less than 30 min when the light dose is equal to 100 µW/cm^2^.

## 3. Materials and Methods

### 3.1. CAM Model Preparation and Luminescence Lifetime Detection

Fertilized chicken eggs (Animalco AG, Staufen, Switzerland) were incubated in an automatic turning incubator (FIEM snc, Buttigliera d’Asti AT, Italy) with the blunt end up for 3 days (37 °C, 65% humidity, 155.4 mmHg atmospheric oxygen pressure). On the 3rd embryo development day (EDD) a small hole (3 mm in diameter) was perforated in the shell and covered by tape (Scotch^®^ Magic^TM^, St. Paul, MN, USA). Eggs were then returned into the incubator with the blunt end down in a static position until a measurement. The hole in the shell was enlarged (2.5 cm in diameter) at EDD 11.

The chicken embryo chorioallantoic membrane (CAM) was placed under an epi-fluorescence microscope (Nikon Eclipse E 600 FN, Nikon, Tokyo, Japan) to visualize and measure luminophores. A physiological solution of 0.9% NaCl (Braun Melsungen AG, Melsungen, Germany) containing 10 mg/kg of body weight (b.w.) of dichlorotris(1,10-phenanthroline)-ruthenium(II) hydrate ([Ru(Phen)_3_]^2+^, 98% purity powder, Sigma-Aldrich, St. Louis, MO, USA) was administered intravenously (iv) with 20 µL aliquots into the main vein of the CAM. The [Ru(Phen)_3_]^2+^ luminescence was detected with a digital scientific camera (PCO.1300, PCO Imaging, Kelheim, Germany) under a Hg-arc lamp (HBO 103 W/2, Osram, Munich, Germany) excitation at 470 ± 20 nm. The emission was separated using a 505 nm dichromatic mirror, and a long-pass emission filter at 520 nm. A low magnification objective (4×/0.13, Plan Fluor ∞/−, Nikon, Tokyo, Japan) was used to visualize the [Ru(Phen)_3_]^2+^ biodistribution. Image analysis was performed with the Image J software (National Institutes of Health, Bethesda, MD, USA).

The luminescence lifetime of 1 mg/kg of b.w. [Ru(Phen)_3_]^2+^ was measured using a dedicated optical fiber-based, time-resolved spectrometer previously described [3,7]. A nitrogen laser-pumped tunable dye (Coumarin 102) laser emitting at 470 nm (<10 ns pulse duration, 10 Hz repetition range) was coupled into a single optical fiber (500 µm diameter) to probe the CAM vessels. The same fiber was used to collect the [Ru(Phen)_3_]^2+^ luminescence filtered by a 660–735 nm emission filter (HQ 700/75 M), and detected by a gateable photomultiplier. Autofluorescence was subtracted. Luminescence decays were measured at different locations on the CAM as presented in Figure 1: in the vessels (‘in’) and out of the vessels (‘out’). The eggs were placed into a gas chamber and subjected to 0, 10, and 20% O_2_ in N_2_ (*w*/*w*) that correspond to pO_2_: 0, 74, and 155.4 mmHg. Pure nitrogen gas (Carbagas, Muri bei Bern, Switzerland) was mixed by the BRICK-gas mixer (Life Imaging Services GmbH, Basel, Switzerland) with air to reach gas mixtures. The [Ru(Phen)_3_]^2+^ luminescence lifetimes were measured at 5, 10, and 20 min after its administration and gas application. Eggs were kept at 37 °C. Ten eggs were measured per condition.

### 3.2. Luminescence Lifetime Determination

**Figure 13:**
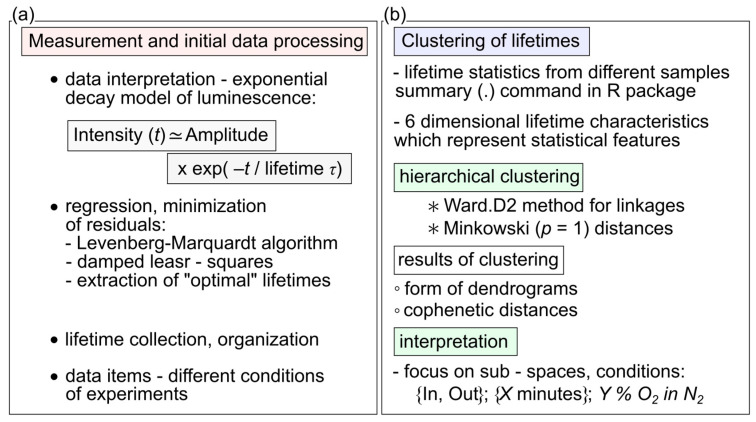
Schematic diagrams of (**a**) measurement and initial data processing, and (**b**) clustering of lifetimes.

Although the clustering problem is the subject of an extensive literature with stringent arguments, we will briefly deal with only a few of its aspects that are relevant for the experimental data application in question. Generally, the clustering approach can be useful in scientific areas where data occur in clumps, which were referred to as clusters. In short, our study can be viewed as a technique where the decays of luminescence intensity are derived (see Figure 1c and Figure 13a) to provide data inputs that the clustering handles further. The next step introduces the set of lifetimes, which is assumed to consist of a system of all lifetimes corresponding to the same conditions of observation. Using approaches known as descriptive statistics, we can better understand the properties of a set of observations. As a next step, we used the summary (.) function in R [33]. The six components: minimum, 1st quartile, arithmetic mean, median, 3rd quartile, and maximum can be calculated for the set of lifetimes corresponding to the given experimental conditions (see Figure 2 and Figure 13b). The components are forming six-dimensional vectors which can be compared in terms of distances or distance-based measures. More specifically, we used Ward’s agglomerative clustering algorithm, which is implemented in R (open-source software for statistical calculations) along with a standard hclust (.) function. The effects were discussed in connection with the Minkowski-type of the distance matrix (in the case of *p* = 1) leading to Ward’s technique [40]. For specificity, we state that using R defined by the Ward.D2 method, the distance matrix was transformed into a system of dendrograms (Figure 3). The cophenetic distance between two objects in the dendrogram is the distance of the objects—vectors that are members of the same cluster. Climbing up the dendrogram tree is described by the Height, which is the normalized lifetime difference referring to the pair of experimental conditions associated with the dendrogram legs.

### 3.3. Luminescence Properties of [Ru(Phen)_3_]^2+^ in Solutions

The [Ru(Phen)_3_]^2+^ UV-Vis absorption spectra in the presence and absence of 1 mM H_2_O_2_ (VWR, Paris, France) and H_2_O_2_ alone diluted in distilled water were detected with an absorption spectrometer (UV-2401 PE, Shimadzu, Sydney, Australia) in the spectral range 200–600 nm with 1 nm step.

The luminescence decays of 10 µM [Ru(Phen)_3_]^2+^ in the 0.9% NaCl solutions in the absence and presence of 200 nM and 1 mM H_2_O_2_ were detected as previously described [32]. The samples were excited at 476 nm by CW laser (90C FreD, Coherent, Santa Clara, CA, USA). The laser beam passed via an acousto-optical modulator (AOM, 1205C, Isomet, Springfield, VA, USA) operated in switching ON/OFF mode. It was driven by electrical pulses from a delay generator (20 µs laser pulse, 2 kHz repetition rate). Gas flow (20% O_2_ in N_2_ (*w*/*w*)) and temperature (25 °C) controllers were embedded in a quartz cuvette. The phosphorescence signal was purified by a long-pass filter (>500 nm) and measured with an avalanche photo-diode (APD, APD110A2, Thorlabs, North Newton, NJ, USA). A mono-exponential fit was applied to derive the [Ru(Phen)_3_]^2+^ luminescence lifetime. The average value was estimated from 10 measurements.

### 3.4. Cell Culture Preparation and Confocal Fluorescence Imaging

The U87 MG human glioma cells (Cells Lines Services, Eppelheim, Germany) were grown in cell culture medium Dulbecco’s modified Eagle medium (D-MEM, Gibco-Invitrogen, Life Technologies Ltd., Paisley, UK) supplemented with 10% fetal bovine serum (FBS), L-glutamine (862 mg/L), sodium pyruvate (110 mg/L), glucose (4500 mg/L) and penicillin/streptomycin (1% *w*/*w*), all from Gibco-Invitrogen, Life Technologies Ltd., Paisley, UK. The cells were incubated in the dark at 37 °C, 5% CO_2_, and 80% humidified atmosphere. Cells were seeded into the glass-bottom embedded Petri dishes (SPL Confocal dish PS/glass hole, 35 × 10 mm^2^) for microscopy at density 10^4^ cells per petri dish.

The cells were labeled with 1 µM CellROX^®^ Green (CellROX, Life Technologies^TM^, Carlsbad, CA, USA) and 400 nM MitoTracker^TM^ Orange CMTMRos (MTO, ThermoFisher Scientific, Waltham, MA, USA) to detect oxidative stress and mitochondrial integrity. Cells were treated with 200 µM [Ru(Phen)_3_]^2+^ and 10 µM curcumin (Sigma-Aldrich, Darmstadt, Germany) for 1 and 24 h. Fluorescence images were collected with a confocal fluorescence microscope system (LSM 700, Zeiss, Oberkochen, Germany), 63X oil immersion objective (NA 1.46, Zeiss), and a CCD camera (AxioCam HRm, Zeiss). The samples were excited with 405, 488, and 555 nm lasers. The emission was detected as follows: CellROX and curcumin 488 nm/500–550 nm, [Ru(Phen)_3_]^2+^ 405 nm/<580 nm, MTO 555 nm/<580 nm. The fluorescence images were analyzed with Zen 2011 software (Zeiss). The oxidative stress was induced by 200 µM H_2_O_2_ administration and by light from the microscope system during detection. The experiments were performed in triplicates. Cells with [Ru(Phen)_3_]^2+^ only and in combination with MTO were irradiated during 30 min with blue light as well.

Viable mitochondria were also stained with 5 µM Rhodamine 123 (Rh123, Sigma-Aldrich, Darmstadt, Germany) for 30 min. The samples were excited with 488 nm laser and the fluorescence was detected in the spectral range 490–530 nm. The nuclei were stained with 10 µg/mL Hoechst 33258 (ThermoFisher Scientific, Waltham, MA, USA) during 30 min. The samples were excited with 405 nm laser and the fluorescence was detected in the spectral range 410–480 nm.

Cellular ROS were determined by 2′,7′-dichlorofluorescin diacetate (DCFDA/H2DCFDA) assay kit (ab113851, Abcam, Cambridge, UK) in cells before and after irradiation, in the presence of [Ru(Phen)_3_]^2+^, and 200 µM H_2_O_2_. The samples were excited with 488 nm laser and the fluorescence was detected in the spectral range 490–530 nm. The quantification of H2DCFDA fluorescence intensity was performed with ImageJ. The mean values of fluorescence intensities (8 images) in the extracellular area and the intracellular area were plotted in histograms. Error bars represent standard deviations. The level of significant difference from the control was calculated with one-way ANOVA: * *p* < 0.05, ** *p* < 0.01, *** *p* < 0.001.

Lipid peroxidation was estimated with the lipid peroxidation assay kit (ab243377, Abcam) in cells in the absence and presence of 200 µM H_2_O_2_. The samples were excited with 488 nm laser and the fluorescence was detected in the spectral ranges 490–530 nm (green channel) and 560–630 nm (red channel). Lipid peroxidation is represented with increasing of green fluorescence.

Quantification of lipid peroxidation sensor fluorescence was performed with a 96-well plate fluorescence reader (GloMax TM-Multi1Detection system with Instinct Software, Madison, WI, USA) with blue (excitation at 490 nm, green emission at 510–570 nm) and green (excitation at 525 nm, red emission at 580–640 nm) filters. Cells were seeded in the wells of 96-well plate at the density 9 × 10^3^ cells per well and treated with H_2_O_2_ and lipid peroxidation sensor similarly as for microscopy. The mean values of the fluorescence (three measurements) are presented as the histograms. Error bars represent standard deviations. The level of significant difference was calculated with one-way ANOVA: * *p* < 0.05, ** *p* < 0.01.

### 3.5. Metabolic Activity of U87 MG Cells before and after Irradiation

The U87 MG cells were treated with 400 nM MTO, 200 µM [Ru(Phen)_3_]^2+^, 10 µM curcumin, and their combinations for 1 h. After 1 h the treated cells were kept in dark and irradiated for 5 and 20 min with home-made blue LED-based irradiation platform at 463 ± 10 nm and 100 µW/cm^2^ light dose rate. Metabolic activity was assessed by cell counting Kit-8 (WST-8, Merck, Darmstadt, Germany) at 3rd hour after the irradiation in living cells according to supplier protocol. The absorption of 96-well plates with treated cells was detected with the reader (GloMax TM-Multi1Detection system with Instinct Software, Madison, WI, USA) at 450 nm. The 3-(4,5-dimethylthiazol-2-yl)-2,5-diphenyltetrazolium bromide (MTT, Sigma-Aldrich, Darmstadt, Germany) was applied to cells 24 h after the irradiation according to supplier protocol. The purple crystals of formazan were dissolved in 100% dimethyl sulfoxide (DMSO, Sigma-Aldrich, Darmstadt, Germany) and measured with the absorption reader at 560 nm.

### 3.6. Fluorescence and Phosphorescence Lifetime Imaging of U87 MG Cells

An inverted fluorescence microscopy system (Zeiss AxioObserver Z1, Zeiss) equipped with 40× water immersion objective (NA = 1.2, Zeiss) and connected to a fluorescence lifetime imaging system (FLIM, DSC-120 Dual Channel Confocal Scanning system, Becker & Hickl GmbH, Berlin, Germany) was used for time-resolved fluorescence microscopy. The 400 nM MTO was detected in the fluorescence lifetime imaging (FLIM) mode. The samples were excited with a pulsed NKT-Super-K Extreme laser (40 MHz, 5 ps pulse, NKT Photonics, Birkerød, Denmark) at 555 nm (2% of total power). The MTO fluorescence lifetimes were detected at the 25 ns time range with an HPM-100-50 hybrid detector (Becker & Hickl GmbH). The 200 µM [Ru(Phen)_3_]^2+^ and 10 µM curcumin were detected in the phosphorescence lifetime imaging (PLIM) mode. Samples were excited with the same laser as for MTO at 470 nm (10% of total power) and a scan rate of 10.23 ms line time, 2.62 s/frame. The [Ru(Phen)_3_]^2+^ and curcumin phosphorescence lifetimes were detected at the 25.6 µs time range with the same HPM-100-50 hybrid detector. The light path was filtered with LP 488 and BP 624 ± 20 nm (NT67-035, EDMUND Optics, Barrington, NJ, USA). Time decays were analyzed with SPC image analysis software (Becker & Hickl GmbH). The quality of the fits was graphically checked by plotting the residuals and χ^2^~1. The experiments were performed in triplicates. Different regions of interest (ROI) were selected. The positions of ROI were depicted in figures. The FLIM of MTO was fitted with a mono-exponential function. The PLIM of [Ru(Phen)_3_]^2+^ were fitted with bi-exponential function and t2 was fixed at 20,000 ns (a probability of the occurrence was below 1). FLIM and PLIM were detected in living cells treated 1 h with MTO, curcumin, and [Ru(Phen)_3_]^2+^. All probes were maintained in the media during detection. Intracellular [Ru(Phen)_3_]^2+^ detection was performed 24 h after the treatment. Oxidative stress in cells was induced by 200 µM H_2_O_2_ and by irradiation with blue LED light (463 ± 10 nm and 100 µW/cm^2^ light dose rate) for 30 and 90 min. Cells with [Ru(Phen)_3_]^2+^ only and in combination with MTO were 30 min irradiated with blue light as well. The detection was performed shortly after the irradiation. The luminescence signal and lifetime of [Ru(Phen)_3_]^2+^ was not detected in the MTO fluorescence detection window, and the phosphorescence of MTO in [Ru(Phen)_3_]^2+^ window was eliminated. Curcumin fluorescence was not detected in the MTO fluorescence window. Curcumin phosphorescence was present in [Ru(Phen)_3_]^2+^ detection window, however, the localization of this signal was clearly defined only within the cells.

### 3.7. Western Blot Analysis

The U87 MG human glioma cells were seeded in 25 cm^2^ flasks at the density 5 × 10^5^ cells. Cells were treated for 30 min with 200 µM H_2_O_2_ before lysis in radioimmunoprecipitation (RIPA) buffer (150 mM sodium chloride, 1% Triton X-100, 0.5% sodium deoxycholate, 0.1% sodium dodecyl sulphate, 50 mM Tris, pH 8). Western blot analysis was performed similarly as described in [15]. Oxidative stress defense cocktail (ab179843, Abcam, Cambridge, UK) was applied to estimate the expression level of catalase, superoxide dismutase 1, thioredoxin, and smooth muscle actin in cells. Anti-β-actin antibody (ab8227, Abcam, Cambridge, UK) was determined as a housing protein. The WesternBreeze chromogenic kit anti- rabbit was purchased from ThermoFisher Scientific. The image analysis of proteins on the membrane was performed with ImageJ software. Optical densities (O.D.) of the bands were detected with ImageJ and analyzed, the normalized O.D. values are the mean values from 4 measurements and are plotted in histograms (down). Error bars represent standard deviations. The level of significant difference was calculated with one-way ANOVA: * *p* < 0.05, ** *p* < 0.01.

## 4. Conclusions

The present manuscript brings a new point of view to interpret the data obtained by time-resolved measurement of the [Ru(Phen)_3_]^2+^ luminescence lifetimes in cells. We have shown that hierarchical clustering, a key element in the processing of data sets reflecting the [Ru(Phen)_3_]^2+^ oxygen sensitivity, offers many opportunities to carry out the analysis. Significant relationships in the tissue oxygenation level detections were revealed by specific differences between dendrograms. This study enabled to show that [Ru(Phen)_3_]^2+^ molecules distributed in the extravascular space were more sensitive to external changes of the oxygen level, the dendrograms unambiguously segmented the data of high and low oxygenated tissues (in space and time). We have assumed that the oxygen sensitivity of [Ru(Phen)_3_]^2+^ luminescence lifetime is influenced by oxidative stress induced in cells. For this reason, and for the simplicity, [Ru(Phen)_3_]^2+^ and MTO were co-administered and their luminescence and fluorescence lifetimes were detected with the unique fluorescence and phosphorescence lifetime imaging microscope to register the changes in the oxygenation and oxidative stress in glioma cells.

With our new approach, we have demonstrated that [Ru(Phen)_3_]^2+^ can be applied in combination with MTO to detect extracellular oxygen and intracellular oxidative stress levels. This combination works well when oxidative stress is induced externally, for example when H_2_O_2_ is used for this purpose. However, PDT which is known to consumed oxygen intracellularly did not induce variations of the [Ru(Phen)_3_]^2+^ luminescence lifetime in cell culture media in our studied system. On the other hand, MTO sensing revealed both, antioxidant and oxidative stress production activity of curcumin-mediated PDT. 

We have demonstrated that the photo-toxicity of the combination of MTO and [Ru(Phen)_3_]^2+^ was low and that the detection of oxygenation and oxidative stress can be performed without cell photodamage when such measurements are performed up to 1 h after the administration of these dyes when the excitation is at 463 ± 10 nm with an irradiance of 100 µW/cm^2^ (30 min).

It would be interesting to see what would happen if this approach is applied to cancer cell spheroids as a model of tumor. One can expect that [Ru(Phen)_3_]^2+^ will localize in the interstitial space, hence probing the oxygen level in spheroids instead of in the cells monolayers. Another advantage of the small 3D system is that the diffusion of oxygen and its consumption will be better controlled in the small volume of the spheroid in comparison with the CAM. Using optimized PS and oxygen-sensitive probes will probably enable to improve the imaging in real-time of the photo-destruction, oxygen consumption, and oxidative stress induction during PDT.

## Figures and Tables

**Table 1:** Additional information on descriptive statistics of [Ru(Phen)_3_]^2+^ luminescence lifetimes corresponding to different experimental conditions in the CAM. To clarify the meaning of the symbols used in the table headings, please refer to their meanings below in Equations (1)–(3). The color code is the same as in dendrograms in Figure 3 and Figure 4.

Regime Data Set	rel_Md_	rel_OI_	Mean *τ* [ns]	% O_2_ in N_2_ Calculated Equation (1)
0% O_2_ in N_2_, 5 min, in	0.029		710.8	10.80
0% O_2_ in N_2_, 5 min, out	0.009	0.032	733.6	9.83
0% O_2_ in N_2_, 10 min, in	0.013		743.1	9.44
0% O_2_ in N_2_, 10 min, out	0.024	0.033	768.4	8.46
0% O_2_ in N_2_, 20 min, in	−0.006		774.3	8.24
0% O_2_ in N_2_, 20 min, out	0.030	0.028	796.6	7.44
10% O_2_ in N_2_, 5 min, in	0.005		626.5	14.99
10% O_2_ in N_2_, 5 min, out	−0.013	0.067	670.4	12.67
10% O_2_ in N_2_, 10 min, in	0.000		616.6	15.55
10% O_2_ in N_2_, 10 min, out	−0.018	0.103	683.3	12.05
10% O_2_ in N_2_, 20 min, in	−0.001		679.6	12.23
10% O_2_ in N_2_, 20 min, out	−0.004	0.002	681.1	12.15
20% O_2_ in N_2_, 5 min, in	0.012		609.0	16.00
20% O_2_ in N_2_, 5 min, out	−0.005	0.039	633.3	14.61
20% O_2_ in N_2_, 10 min, in	0.008		624.9	15.08
20% O_2_ in N_2_, 10 min, out	0.012	0.041	651.3	13.64
20% O_2_ in N_2_, 20 min, in	0.005		644.6	13.99
20% O_2_ in N_2_, 20 min, out	−0.011	0.006	648.5	13.79

## Data Availability

Data contained in this paper are available from the authors.

## Supplementary Materials

The following are available online, Figure S1: Decomposition of confocal microscopy images of U87 MG cells stained with CellROX^®^Green, rhodamine 123, and [Ru(Phen)_3_]^2+^; Figure S2: Mitochondria of U87 MG cells in the presence and absence of H_2_O_2_; Figure S3: Western blot analysis of oxidative stress defense proteins: catalase, superoxide dismutase 1, and thioredoxin in U87 MG cells in the absence and presence H_2_O_2_; Figure S4: Cellular ROS visualized with DCFDA/H2DCFDA assay in U87 MG cells in the presence of [Ru(Phen)_3_]^2+^, light and H_2_O_2_; Figure S5: Lipid peroxidation visualized in U87 MG cells in the absence and presence H_2_O_2_; Figure S6: Mitochondria of U87 MG cells in the presence of [Ru(Phen)_3_]^2+^ after irradiation; Figure S7: PLIM images of [Ru(Phen)_3_]^2+^ in U87 MG cells after irradiation; Figure S8: FLIM and PLIM images of MTO and [Ru(Phen)_3_]^2+^ before and after irradiation of U87 MG.

## Sample Availability

Samples of the compounds are commercially available.
