# Supplementary material for: Influence of Oxidative Stress on Time-Resolved Oxygen Detection by [Ru(Phen)3]2+ In Vivo and In Vitro
Source: Molecules. 2021 Jan 18;26(2):485. doi: 10.3390/molecules26020485 (PMC7831141; doi:10.3390/molecules26020485)
Supplement: Supplementary file 1 [file molecules-26-00485-s001.pdf]

Supplementary Materials

Influence of Oxidative Stress on Time-Resolved Oxygen Detection by $[Ru(Phen)_3]^{2+}$ In Vivo and In Vitro

Veronika Huntosova [1,*], Denis Horvath [1], Robert Seliga [1] and Georges Wagnieres [2]

[1] Center for Interdisciplinary Biosciences, Technology and Innovation Park, P.J. Safarik University in Kosice, Jesenna 5, 041 54 Kosice, Slovakia; denis.horvath@upjs.sk (D.H.); robert.seliga@upjs.sk (R.S.)
[2] Laboratory for Functional and Metabolic Imaging, Institute of Physics, Swiss Federal Institute of Technology in Lausanne (EPFL), Station 6, Batiment de Chimie, 1015 Lausanne, Switzerland; georges.wagnieres@epfl.ch
* Correspondence: veronika.huntosova@upjs.sk; Tel.: +421-55-243-2243

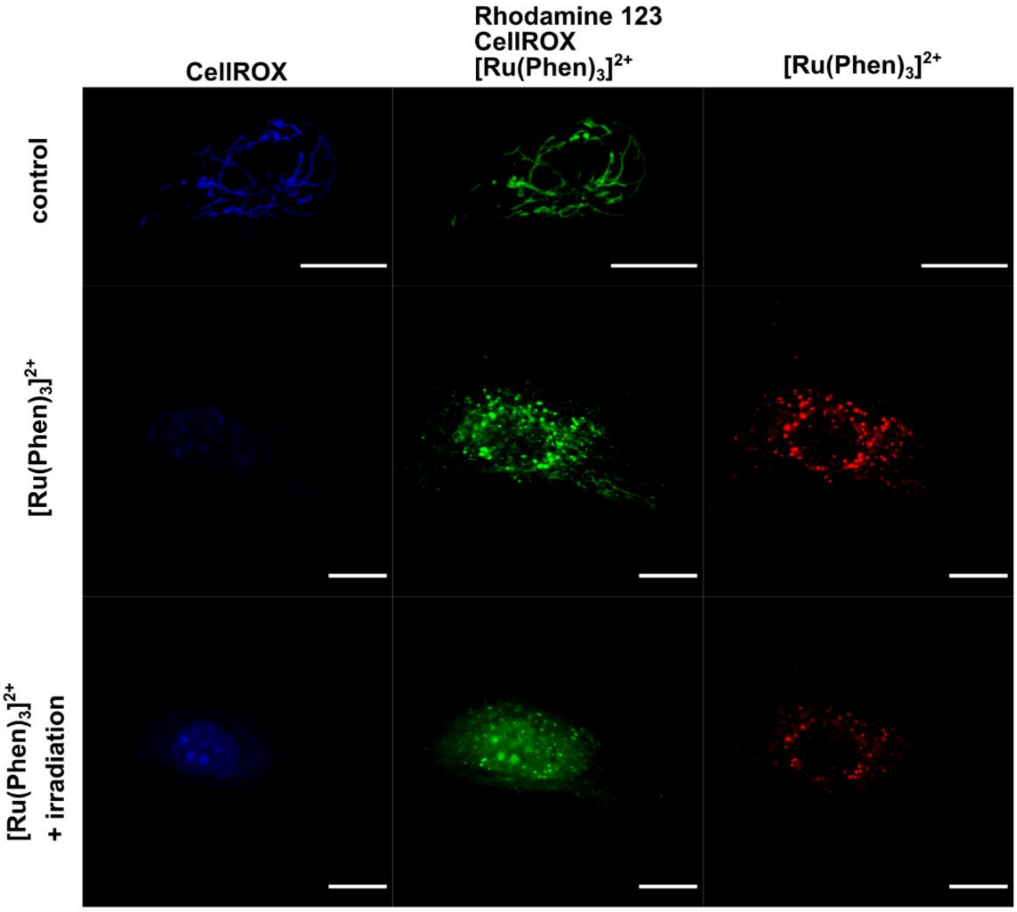

Figure S1. Decomposition of confocal microscopy images of U87 MG cells stained with 1 μM (30 min) CellROX®Green (blue and green channel), 5 μM rhodamine 123 (green channel) and 200 μM $[Ru(Phen)_3]^{2+}$ (green and red channel). The cells were irradiated with microscopy (detection time less than 2 min, 2% of the power of the laser: 405 and 488 nm)—the second scan of cells stained with $[Ru(Phen)_3]^{2+}$, rhodamine 123 and CellROX®Green. The photoreaction induced oxidative stress discovered by fluorescence in the nucleus of the cell. While in control cells CellROX®Green was mostly localized in the mitochondria, in damaged cells it was localized in the nucleus. The scale bars represent 20 μm.

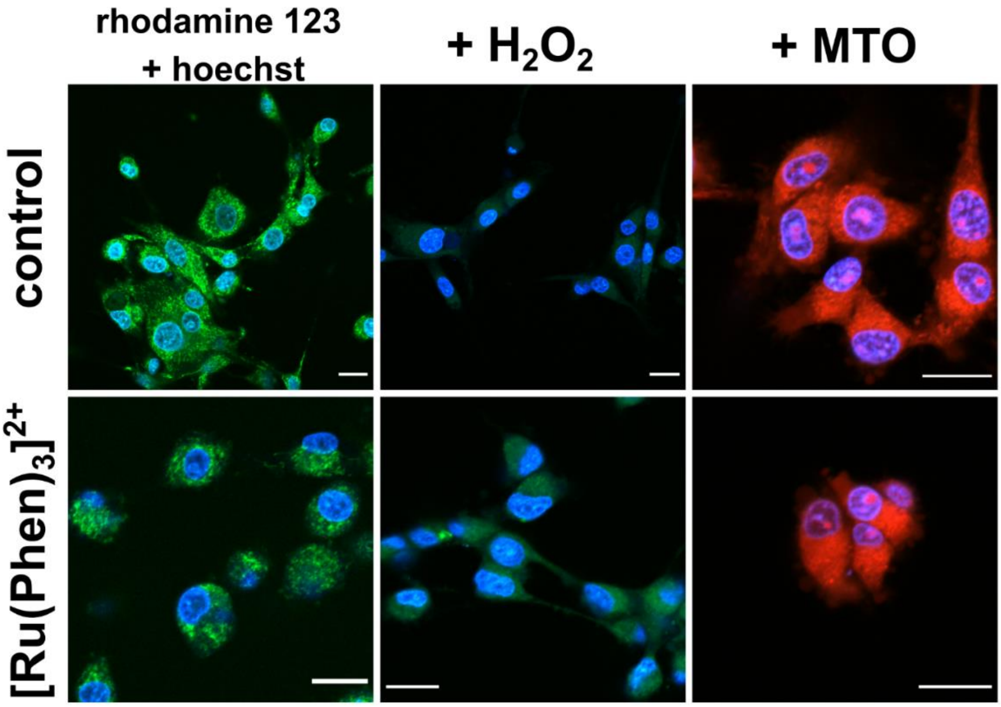

Figure S2. Fluorescence images of U87 MG cells in the presence (30 min) and absence of 200 μM H_2O_2. Control and cells in the presence of $[Ru(Phen)_3]^{2+}$ were labeled with rhodamine 123 (green) and MTO (red). Rhodamine 123 is sensitive to mitochondrial membrane potential. It is localized in the mitochondria of live cells, and its fluorescence drops down with dissipation of mitochondrial membrane potential (diffused localization). MTO persists to be localized in mitochondria of damaged cells after H_2O_2 application. Granular mitochondria can be observed. The nuclei of the cells were counterstained with Hoechst (blue). The scale bars represent 20 μm.

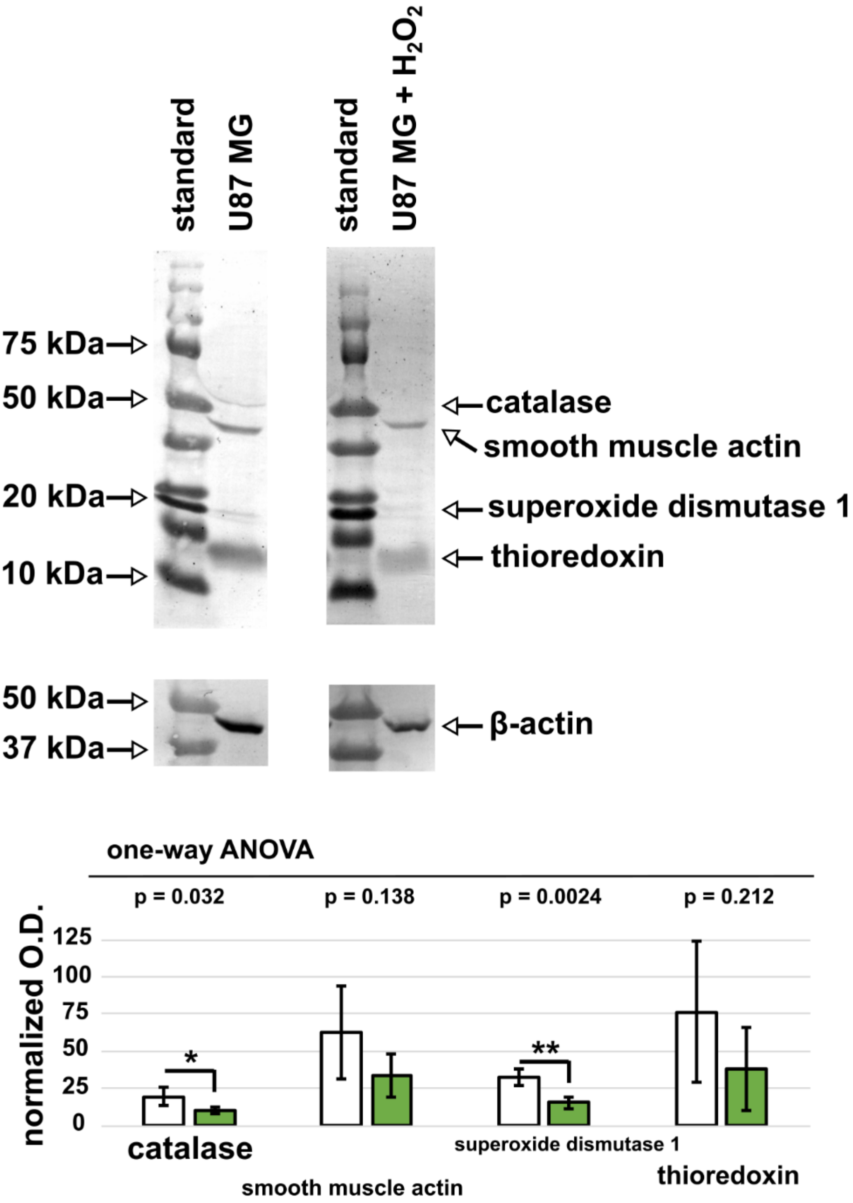

Figure S3. Western blot analysis of oxidative stress defense proteins: catalase, superoxide dismutase 1, and thioredoxin in 87 MG cells in the absence and presence (30 min) of 200 μM H_2O_2. Smooth muscle actin and β-actin were detected as the loading controls. Optical densities (O.D.) of the bands were detected with ImageJ and analyzed, the normalized O.D. values are the mean values from 4 measurements and are plotted in histograms (down). Error bars represent standard deviations. The level of significant difference was calculated with one-way ANOVA: $*p < 0.05$, $**p < 0.01$. Catalase and superoxide dismutase 1 decreased in cells treated with H_2O_2.

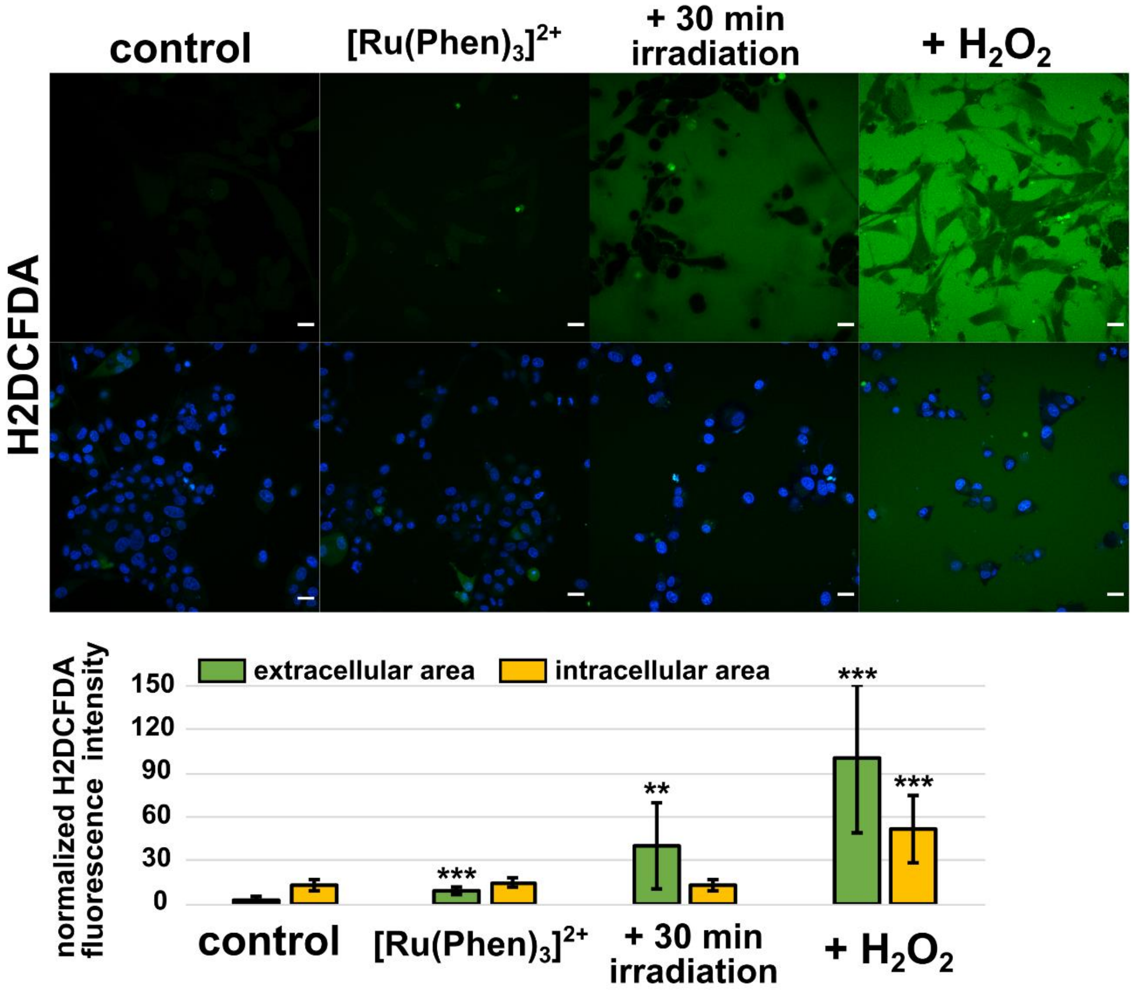

Figure S4. Fluorescence images of U87 MG cells in the presence and absence of $[Ru(Phen)_3]^{2+}$ before and 30 min after the irradiation with blue light. Positive control was treated with 200 µM H_2O_2. Cellular ROS were visualized with DCFDA/H2DCFDA assay (green). The nuclei of the cells were counterstained with Hoechst (blue). The scale bars represent 20 µm. The quantification of H2DCFDA fluorescence intensity was performed with ImageJ. The mean values of fluorescence intensities (n = 8 images) in the extracellular area (green column) and the intracellular area (yellow column) were plotted in histograms (down). Error bars represent standard deviations. The level of significant difference from the control was calculated with one-way ANOVA: *$p < 0.05$, **$p < 0.01$, ***$p < 0.001$. ROS significantly increased in the extracellular area after treatments. Significant extracellular and intracellular ROS were detected in cells after H_2O_2 administration—green fluorescence increased. We present fluorescence images with and without Hoechst staining because Hoechst partially affects green DCFDA/H2DCFDA fluorescence detection.

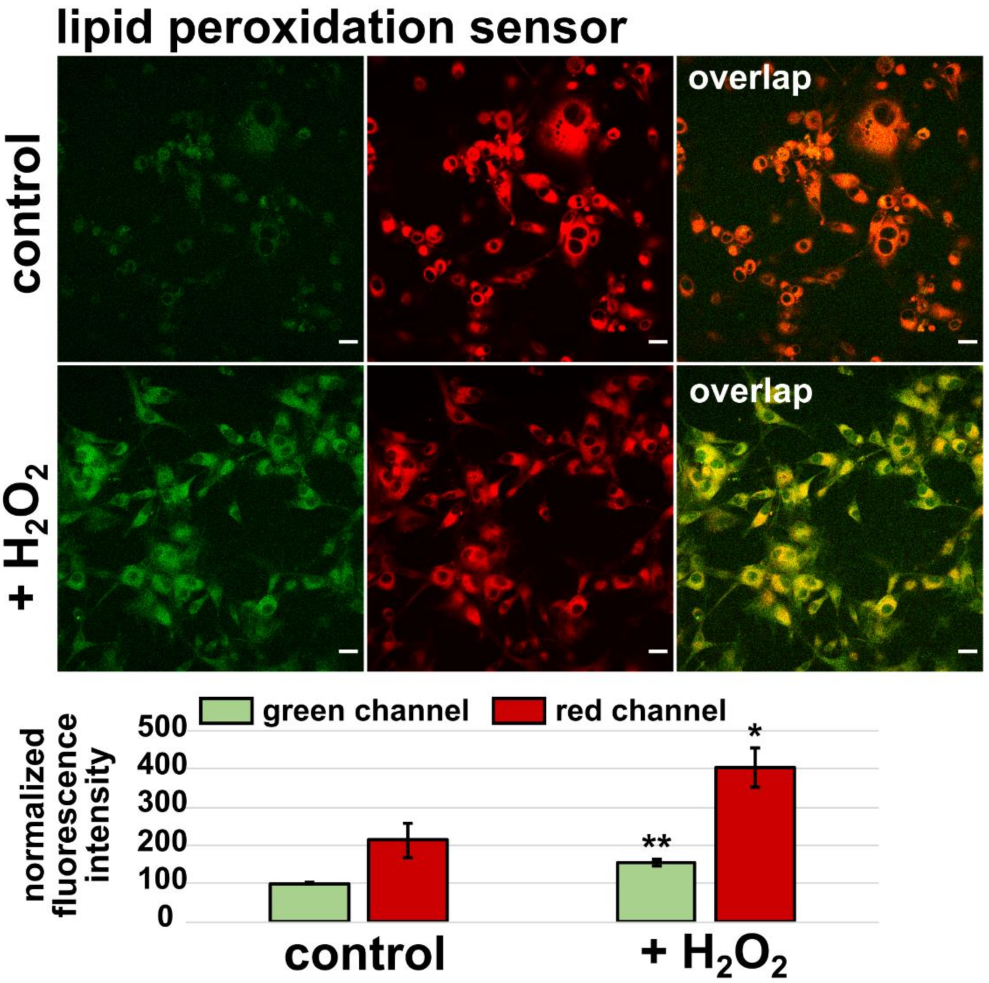

Figure S5. Fluorescence images of U87 MG cells in the presence (30 min) and absence of 200 μM H_2O_2. Lipid peroxidation was visualized with a lipid peroxidation sensor (red/green). The scale bars represent 20 μm. Quantification of lipid peroxidation sensor fluorescence was performed with a 96-well plate fluorescence reader in green and red channels. The mean values are presented as the histograms (down). Error bars represent standard deviations. The level of significant difference was calculated with one-way ANOVA: *$p < 0.05$, **$p < 0.01$. H_2O_2 induced lipid peroxidation in cells accompanied by increasing green fluorescence. The overlap of green and red channels is more yellowish/green. Fluorescence intensities significantly increased after H_2O_2 administration in both spectral ranges.

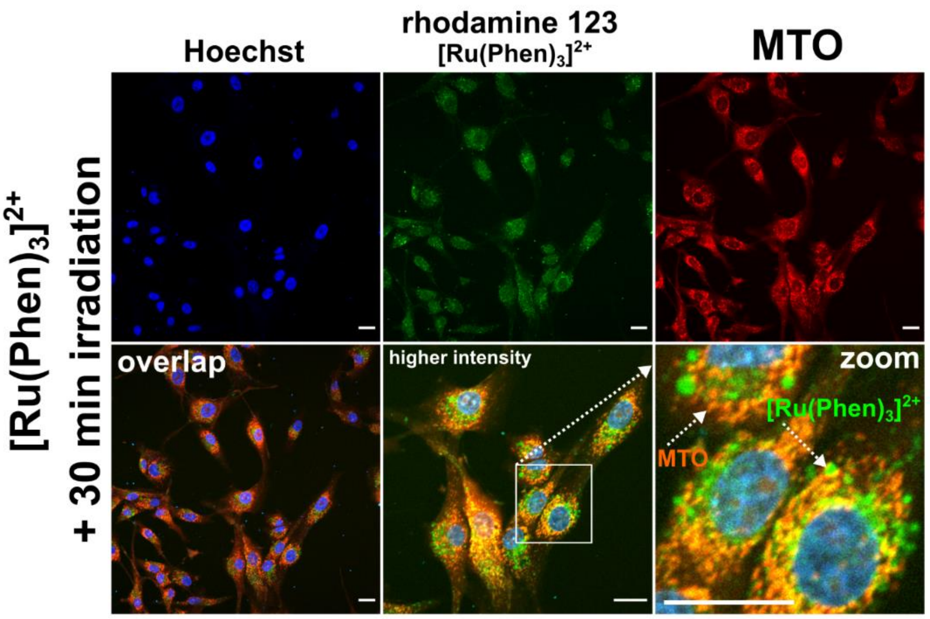

Figure S6. Fluorescence images of U87 MG cells in the presence of $[Ru(Phen)_3]^{2+}$ and 30 min after the irradiation with blue light. Cells were labeled with rhodamine 123 (green) and MTO (red). $[Ru(Phen)_3]^{2+}$ signal was in the same channel as rhodamine 123. Mitochondria of cells labeled with MTO become swollen (red/orange). The nuclei of the cells were counterstained with Hoechst (blue). The scale bars represent 20 µm. The intensity was increased to better distinguish MTO and $[Ru(Phen)_3]^{2+}$ emission. $[Ru(Phen)_3]^{2+}$ was recognized in the extracellular area and in the vesicles (green round organelles) within the cells.

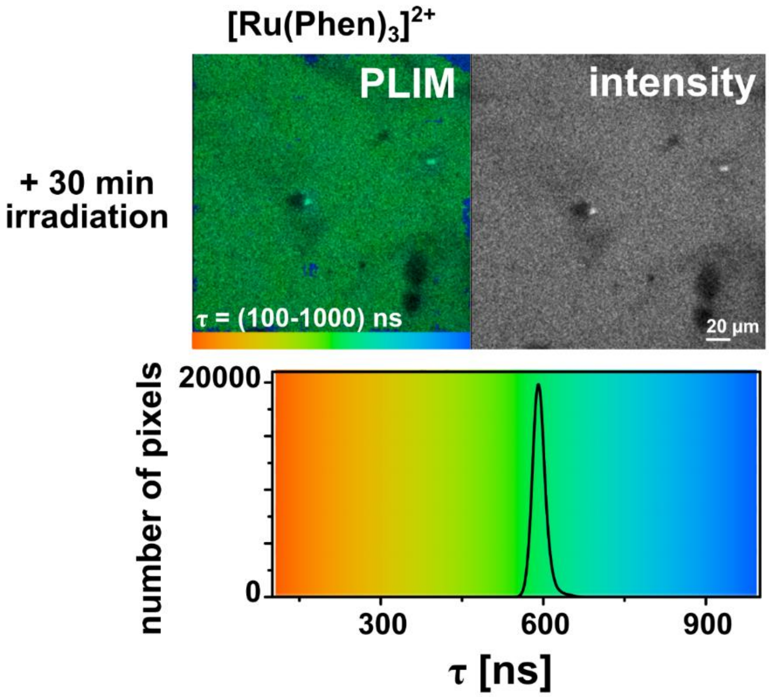

Figure S7. Illustrative intensity and PLIM images obtained with 200 μM $[Ru(Phen)_3]^{2+}$ applied in the culture medium of U87 MG cells for 1 h. PLIM was detected 30 min after the irradiation of cells with blue light. Down: $[Ru(Phen)_3]^{2+}$ luminescence lifetime distribution histograms. The luminescence lifetimes are color-coded (100 ns—red, 1000 ns—blue). The samples were excited with laser light at 470 nm. The scale bars represent 20 μm.

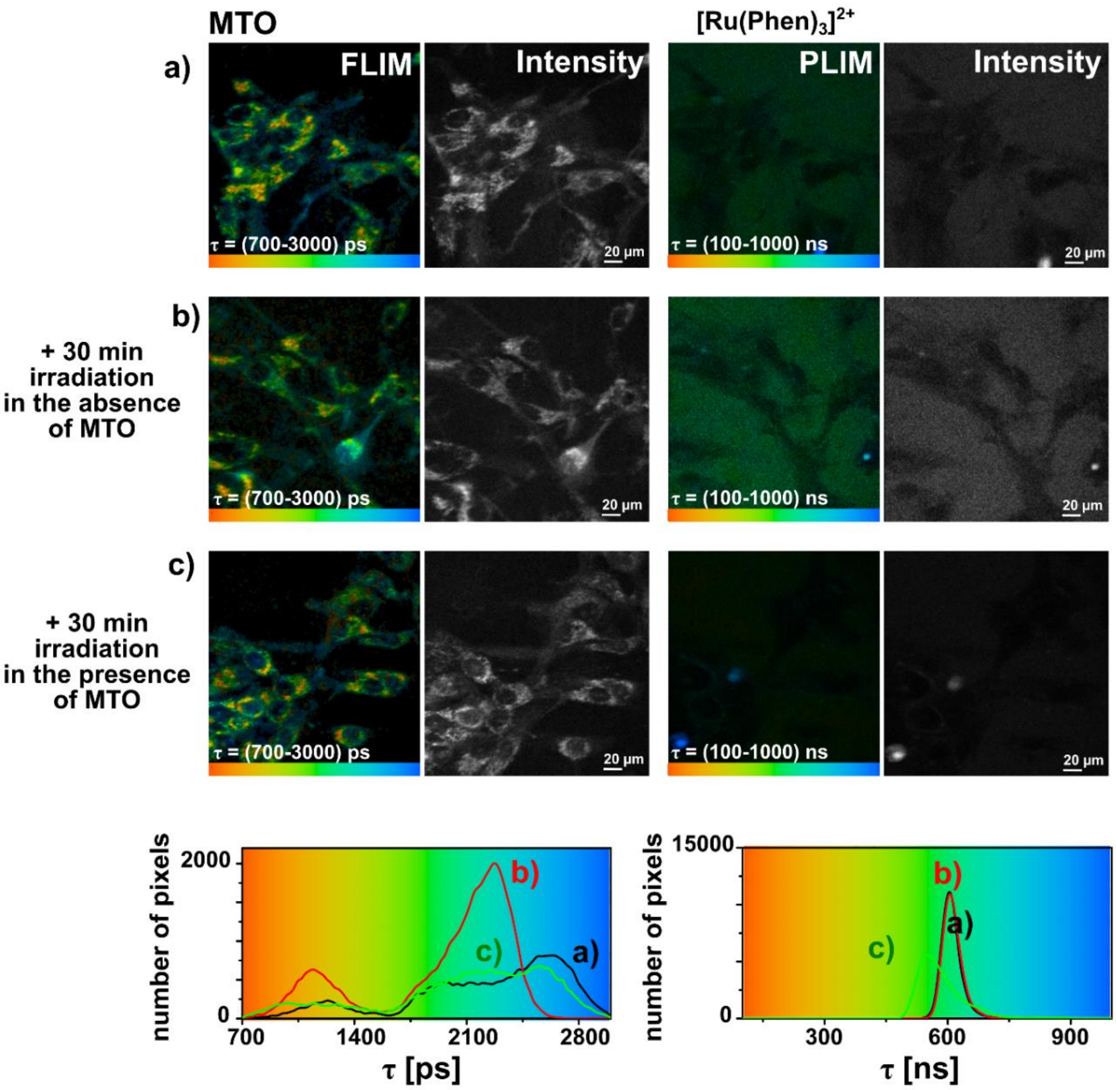

Figure S8. Representative FLIM and PLIM images of MTO (left) and $[Ru(Phen)_3]^{2+}$ (right) applied in the medium of U87 MG cells (**a**) in the absence and (**b**,**c**) presence of irradiation with blue light. MTO was (**c**) present or (**b**) not in the medium during the irradiation. Down: MTO and $[Ru(Phen)_3]^{2+}$ luminescence lifetime distribution histograms. The luminescence lifetimes are color-coded (minima—red, maxima—blue). Gray-scaled images represent the luminescence intensity of MTO and $[Ru(Phen)_3]^{2+}$. The samples were excited with laser light at 555 nm (MTO) and 470 nm ($[Ru(Phen)_3]^{2+}$). The scale bars represent 20 μm.
